# Monitoring the implementation and scale-up of a life-saving intervention for preterm and small babies: Facility-based Kangaroo Mother Care

**DOI:** 10.7189/jogh.11.14001

**Published:** 2021-07-24

**Authors:** Laura Marcela Torres, Goldy Mazia, Tanya Guenther, Bina Valsangkar, Steve Wall

**Affiliations:** Save the Children, Washington, DC, USA

## Abstract

**Background:**

Kangaroo mother care (KMC) is an evidence-based intervention with large protective effects on neonatal mortality and morbidity, especially among small babies. Despite the available evidence, KMC adoption, implementation and scale-up has lagged. The purpose of this paper is to inform current and future KMC implementation by identifying achievements and challenges in countries that are in the process of scaling up KMC.

**Methods:**

We collected and analyzed information to track the status of facility-based KMC in countries identified by the KMC Acceleration Partnership. We assessed the status of the scale-up in six priority countries (Ethiopia, Malawi, Nigeria and Rwanda in Africa, and Bangladesh and India in Asia) for three periods: 2014 and prior, 2015-2017 and 2017-2019 across six strategic areas: national policy, country implementation, research, knowledge management, monitoring and evaluation and advocacy. We collected information through in-depth interviews with key participants, quantitative data extraction from the Demographic Health Survey and secondary data extraction from policies, briefs, program reports and other documents.

**Results:**

Progress in terms of national policy and advocacy appeared to occur quite quickly and evenly across the six priority countries, despite being at different stages during the first assessment. In the areas of country implementation support and research, progress occurred more slowly and results were more variable across countries. It was noted that the number of health facilities offering KMC services increased in all six priority countries, but coverage of KMC was difficult to estimate, demonstrating the ongoing challenges in the area of monitoring and evaluation despite progress made in integrating KMC indicators into national health information systems in five countries. Among the six priority countries – Malawi and Bangladesh had fully achieved at least four the first time six conditions were introduced.

**Conclusions:**

We documented notable achievements in the dimensions of policy and country implementation across the six countries, which were likely driven by government engagement to prioritize newborn care services and the promotion of KMC as a core intervention for small babies. We noted challenges in critical areas such as ambulatory KMC, follow-up, and monitoring and evaluation. Addressing these gaps while securing funding to allocate human resources adequately, promoting acceptance of KMC for demand creation and facilitating the use of data for decision making will be vital to ensure effective coverage at scale.

In the last three decades, notable milestones in decreasing child mortality have been achieved. Between 1990 and 2018, deaths among infants were reduced by 52% (from 37 deaths per 1000 births in 1990 to 18 in 2018), while overall deaths among children under-five dropped by 59% (from 93 deaths per 1000 births to 39) [1]. Despite these reductions, many children continue to die, most of them from preventable causes. Global commitments such as the United Nations Millennium Declaration and the 2030 Agenda for Sustainable Development have prioritized child survival. Reducing under-five mortality rate by two thirds by 2015 was one of the targets in the Millennium Development Goals (MDGs), and even though progress was made, the target was not met [2]. Following the MDGs, the Sustainable Development Goals set a target to end preventable deaths among newborns and to reduce neonatal mortality to 12 per 1000 live births by 2030 [3]. This represents an ideal but high target that calls for the immediate adoption and scale-up of life-saving interventions.

The first 28 days of life are critical to a child’s survival. Every year, an estimated 2.5 million newborns die during this period [4]. Of those who die, about 80% are born with low birth weight (LBW) and two thirds are born prematurely [4]. Moreover, most preterm births occur in Sub-Saharan Africa and Asia (over 80%) [5] and most LBW infants (over 96%) are born in developing countries [6,7], adding to the health burden in already scarce-resource regions [6,7]. Nonetheless, there are evidence-based interventions such as kangaroo mother care (KMC) that reduce the risk of mortality by improving the health outcomes of preterm and/or LBW infants [8,9]. The World Health Organization (WHO) strongly recommends that preterm and full-term newborns weighing 2000 g or less, referred to as small babies, be initiated in KMC at health care facilities (facility-based KMC) as soon as they are clinically stable [10]. KMC is defined as an intervention with different components including prolonged skin-to-skin contact and exclusive breastfeeding [11]. It also includes early hospital discharge, tracking of growth and development and prompt identification and response to illness through follow-up visits [12].

KMC has a large protective effect on neonatal mortality and morbidity[9], as it is associated with a 40% lower risk of mortality at the time of discharge and a 30% lower risk of mortality at the latest follow-up than conventional care [13]. Some positive outcomes directly associated with survival among small babies on KMC are weight gain, earlier discharge, increased likelihood of exclusive breastfeeding [13,14] and long-lasting protective social and behavioural effects that extend well into childhood and beyond [15]. KMC also protects small babies against sepsis, hypothermia, hypoglycaemia and hospital readmission [13,14]. Despite the evidence, KMC adoption, implementation and scale-up have lagged. Multi-pronged strategies are needed to increase global commitment and government buy-in to increase availability, quality and wide-scale uptake of KMC services.

For over two decades after the first documented use of KMC, global agencies have advocated for the scale-up of KMC, but with little discernible progress. WHO and UNICEF launched the Every Newborn Action Plan (ENAP) in 2014, a road map of strategic actions to end preventable newborn deaths. KMC was included in the ENAP as a high-impact, cost-effective intervention to improve newborn health and survival [16]. Other efforts included a call to action in 2013 to accelerate the uptake of KMC, which was the result of a consensus among stakeholders from government, non-governmental organizations, and UN agencies at the KMC Acceleration Convening in Istanbul. The call to action resulted in an ambitious target of reaching “50% coverage of KMC among preterm newborns by the year 2020 as part of an integrated reproductive, maternal, newborn, child health (RMNCH) package” [17]. The KMC Acceleration Partnership (KAP) was established to facilitate accelerated scale-up of facility-based KMC [17]. First, the KAP followed a systematic process to identify countries where KMC had the potential to be accelerated successfully among the 75 countdown countries [1]. Countries were ranked based on leadership and governance, health financing, health information, health service delivery, health workforce, essential supplies and community ownership and partnership. Seven countries were identified as priority countries (Ethiopia, Malawi, Nigeria and Rwanda in Africa, and Bangladesh, India and Indonesia in Asia) and 18 countries were identified as countries of interest (**Figure 1**). Three assessments were conducted to track progress, achievements and limitations during KMC adoption and scale-up efforts for three periods: 2014 and prior, 2015-2017 and 2017-2019.

**Figure 1:**
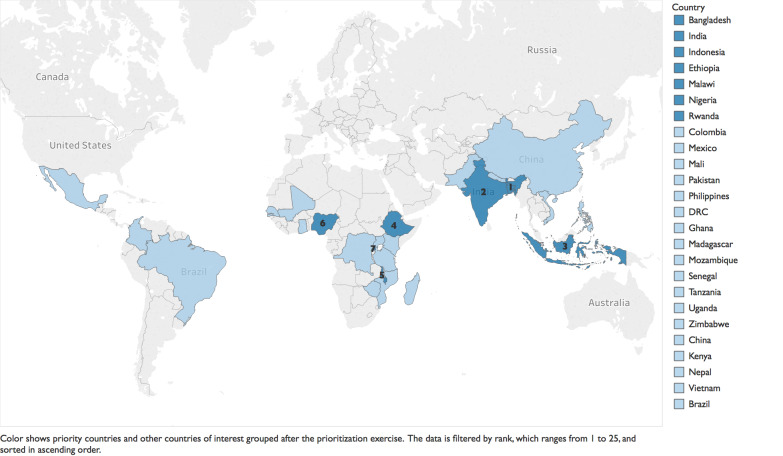
Countries identified in the prioritization exercise.

The purpose of this paper is to describe the results of the three assessments conducted among six of the seven priority countries to inform current and future KMC program implementation.

## METHODS

We conducted three assessments for three periods: 2014 and prior, 2015-2017 and 2017-2019 to collect information about the status of facility-based KMC in six priority countries: Ethiopia, Malawi, Nigeria, Rwanda, Bangladesh and India. We also collected information on eight countries of interest: China, Mali, Mozambique, Pakistan, Philippines, Uganda, Vietnam and Tanzania; and the Dominican Republic. We focused the assessments on six strategic areas: national policy, country support/implementation, research, knowledge management, monitoring and evaluation and advocacy **(****Table 1****)** following the framework developed by the KAP [18].

**Table 1:** Strategic areas from the KMC Acceleration Partnership (KAP)

**National policy**	National health policy
	National guidelines
**Country support/** **implementation**	Levels and types of facilities implementing KMC
	Percentage of LBW newborns initiated in facility-based KMC
	Funding
**Research**	Major or program-based studies currently being conducted related to KMC
**Knowledge management**	Centers of excellence or state-of-the-art facilities for KMC/care of LBW babies
	KMC manuals, trainings and campaigns
**Monitoring and evaluation**	KMC indicators included in the national HMIS
	KMC data recorded at health facilities
**Advocacy**	Professional organizations that endorse KMC
	Awareness campaigns
	Champions

KMC – Kangaroo Mother Care, LBW – low-birth weight

For the six priority countries, data were collected through a 4-step process that included one round of in-depth questions with key participants; extraction of quantitative data from the Demographic Health Survey (DHS), if available; a second round of interviews with open-ended questions to validate the information collected; and secondary data extraction from policies, briefs, program reports and other similar documents. Information was also collected on the status of KMC activities in eight countries of interest. Key participants from these countries were asked over e-mail to fill out a questionnaire (Tables S1 in the **Online Supplementary Document**). Data collected were reviewed along with policy documents, guidelines and scientific research. As we collected information during the 2017 assessment, the information collected from 2014 was further supplemented for all countries. In the assessment conducted in 2019, key participants were asked by e-mail to fill out a questionnaire similar to that used in 2017 but that included follow-up questions regarding pending activities.

***Step 1: Initial in-depth interviews:*** We identified key participants (in-country clinicians, professors and programmatic staff) who had been involved in KMC activities in each country for the first round of in-depth interviews. We contacted them via e-mail requesting participation in the assessment, and we later scheduled a Skype call to go over the questionnaire (Table S1 in the **Online Supplementary Document**). On the day of the interview, participants were asked to consent to participate and to have the interview recorded. Interviews lasted an average of 1 hour and 5 minutes. Interviewees were asked to e-mail documents to support the information given during the interview. Policy documents, studies and manuals were also gathered from government and other relevant websites (see Step 3).

***Step 2: Data extraction from DHS:*** Quantitative data on proxy indicators for KMC (**Table 2**) such as low birth weight, initial breastfeeding and skin-to-skin contact were collected using the information from the latest DHS available for each country.

**Table 2:** Proxy indicators for KMC from Demographic and Health Surveys

**Identification of LBW babies**	Percentage distribution of live births in the 3 y preceding the survey by mother’s estimate of baby’s size at birth, according to background characteristics*
	Percentage of births with a reported birth weight*
	Percentage of babies weighing less than 2.5 kg among births with a reported birth weight*
**Initial breastfeeding indicators**	Percentage of children born in the past 2 y who started breastfeeding within 1 h of birth*
	Percentage of children born in the past 2 y who started breastfeeding within 1 d of birth
**Skin-to-skin contact indicator**	Percentage of births that had skin-to-skin contact immediately following delivery, for women’s most recent live birth in the 3 y preceding the survey*

LBW – low birth weight

*DHS indicators that aim to assess newborn practices right after birth.

***Step 3: Secondary data extraction through document review:*** The information collected from the first round of in-depth interviews and the DHS was outlined in country profiles according to the period and the strategic area. Secondary data extraction from policy, programmatic and other documents was used to support the information collected through the interviews.

***Step 4: Validation and clarification interviews:*** A round of interviews was conducted with key participants based in the United States to clarify, validate and confirm the information presented in the country profiles. Six interviews were conducted, lasting an average of 30 minutes. Drafts of the country profiles were shared with all the key participants following validation during the KMC Acceleration Partnership Community of Practice Meeting in Blantyre, Malawi on October 24-26, 2017 with MOH, implementers and partners from each country. The final draft of the country profiles with the midline assessment was submitted to the respective MOHs for approval. The MOHs of Bangladesh, Ethiopia, Malawi, Nigeria and Rwanda approved the information in the country profiles.

For the third assessment conducted in 2019, key participants were given a structured questionnaire based on the questionnaire used in previous assessments (Table S2 **Online Supplementary Document**). The third assessment’s questionnaire was tailored to each country based on the information provided in the 2017 assessment. Most of the same key participants who were contacted in 2019 had provided information in the 2014 and 2017 assessments.

### Data analysis and use

The information from the three assessments was evaluated against a 1-4 scale (**Table 3**) in the six strategic areas to identify progress and trends.

**Table 3:** Strategic areas with the evaluation scale

**National policy**
1 No national KMC policy or guidelines at the national level.
2 KMC guidelines developed, but no written national policy.
3 KMC guidelines developed. A national policy has been written but not yet approved.
4 National policy and guidelines for KMC in place at the national level.
**Country support/implementation**
1 KMC activities limited to some health facilities. Only donor/international partner funding available.
2 KMC activities offered in multiple health facilities but not yet at a national health facility level. Donor/international partner funding available and some funding available from government/MOH.
3 KMC activities offered in multiple health facilities and expected at all health facilities of one or more national levels. Combination of donor and government/MOH funding available.
4 KMC activities offered at all health facilities of one or more national levels. Combination of donor and government/MOH funding available.
**Research**
1 No major studies and zero or a few program-based studies being conducted related to KMC.
2 Some program-based studies being conducted related to KMC.
3 One major study and some program-based studies being conducted related to KMC.
4 Multiple major studies and program-based studies being conducted related to KMC.
**Knowledge management**
1 No centers of excellence or state-of-the-art facilities for KMC have been established. KMC manuals and trainings not yet developed.
2 One center of excellence or state-of-the-art facility for KMC has been established. KMC manuals and trainings have not been developed.
3 One or more centers of excellence or state-of-the-art facilities for KMC have been established. KMC manuals and trainings have been developed or are in development, but they have not yet been approved.
4 Multiple centers of excellence or state-of-the-art facilities for KMC have been established. KMC manuals and trainings have been developed and approved.
**Monitoring and evaluation**
1 No KMC indicators have been developed. KMC data are not recorded at health facilities.
2 KMC indicators have been developed or are in development. KMC data are not recorded at health facilities.
3 KMC indicators have been developed but have not been included in the national HMIS. KMC data are recorded at health facilities.
4 KMC indicators have been developed and included in the national HMIS. KMC data are recorded at health facilities.
**Advocacy**
1 No known local champion(s). No professional organizations have endorsed KMC.
2 Local champion(s) exist. No professional organizations have endorsed KMC.
3 Local champion(s) exist. One or more than one professional organization has endorsed KMC.
4 Local champion(s) exist. More than one professional organization has endorsed KMC and has actively promoted KMC through trainings, mentorship, and/or the development of guidelines/materials.

KMC – Kangaroo Mother Care, HMIS – health management information system, MOH – Ministry of Health

### Ethical considerations

The procedures and the semi-structured questionnaire for the midline assessment were submitted to the IRB of George Washington University for review. The IRB issued a waiver of documentation of consent because the research presented no more than minimal risk of harm to participants. A consent statement for exempt research, which explains the purpose of the assessment, was shared with all key participants. All key participants who were interviewed gave verbal consent to participate in the assessment and consented to being audio-recorded. The recordings were not transcribed, but they were used as a reference to confirm notes taken during the interviews.

Software used: MS Word (Microsoft Inc, Seattle WA, USA), MS Excel (Microsoft Inc, Seattle WA, USA), Tableau (Salesforce, Mountain View CA, USA), SAS (Cary North Carolina, USA).

## RESULTS

The number of individuals consulted per country during each time period ranged from one to four and included a mix of country-based and global informants. In the 2014 assessment, 31 individuals (22 country-based and nine global) were consulted. In 2017, 26 participants were interviewed either via Skype or in person (17 country-based and nine global). In 2019, the assessment was conducted using structured questionnaires tailored for each country, with 12 participants from 12 countries who responded via e-mail, providing information about the status of KMC activities between 2017 and 2019. A total of nine individuals (four country-based and five global) participated in all three rounds of data collection. The participants represented implementers and partners such as Save the Children and JSI, teaching organizations and the MOHs from various countries. Quantitative data on proxy indicators for KMC were extracted from the most recent DHS for each country. Over 90 policy, educational, programmatic and other documents were reviewed for secondary data extraction.

**Figure 2:**
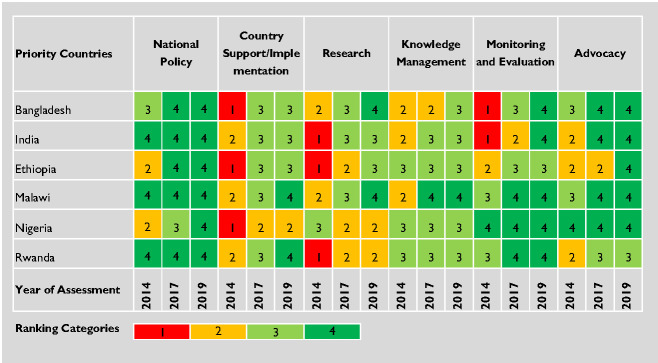
Kangaroo Mother Care (KMC) adoption and implementation assessed and scored in six priority countries.

Detailed information collected for six priority countries (Ethiopia, Malawi, Nigeria, Rwanda, Bangladesh and India) was organised into country profiles (Tables S3 in the **Online Supplementary Document**) and evaluated against the 1-4 scale by strategic area (**Figure 2**). Notable achievements for each of the six priority countries were summarized (**Table 4**). For the eight countries of interest (China, Mali, Mozambique, Pakistan, Philippines, Uganda, Vietnam and Tanzania) and the Dominican Republic, the information was summarised in tables (Tables S4 in the **Online Supplementary Document**).

**Table 4:** Notable achievements across the six priority countries by strategic area between 2014 and 2019

**Bangladesh**	
National policy	KMC is included in the National Newborn Program
	There are national KMC guidelines, a KMC training manual, and monitoring tools
	KMC services are offered at 167 health facilities which represents 62% of the target set by the Ministry of Health and Family Welfare (MOHFW) to be reached by June 2022
Research	Research on KMC implementation in rural areas, lessons learned and implementation research on KMC services were published
Monitoring and evaluation	A monthly KMC reporting form was developed for incorporation into DHIS2
	Five core KMC indicators, including one on follow-up, are listed in the DHIS2
	A KMC database for the DGHS facility was developed
Advocacy	Technical working groups are actively engaged in the development of KMC guidelines and protocols
	Several professional organizations have endorsed KMC
**India**	
National policy	KMC is included in the India Newborn Action Plan (INAP)
	There are national KMC guidelines in place
	Facility and community guidelines for KMC have been included in training packages for health personnel
Monitoring and evaluation	Data on six KMC indicators are reported monthly by 782 health facilities via an online tracking system
Advocacy	KMC is listed in the handbook for community health workers
	KMC is promoted by professional organizations at sessions in conferences and workshops.
	KMC guidelines and communication tools have been developed by professional organizations
	A KMC organization was formed for awareness and advocacy.
**Ethiopia**	
National policy	KMC is part of the national strategy to improve newborn health and child survival
	KMC is covered in the national health plan which aims to improve equity, coverage and utilization of health services, and improve quality of health care
	KMC was prioritized as part of the national strategy to improve health care quality
Advocacy	KMC is actively promoted by the Ethiopian Paediatric Society which has equipped KMC spaces and has trained health workers
**Malawi**	
National policy	KMC was integrated into national health policies
	KMC is part of the maternal and newborn care training package
Country support/ implementation	KMC is offered in the majority of public hospitals
	The establishment of sick newborn care units in all the district hospitals is ongoing
Research	There are several studies being conducted on KMC about different topics on early outcomes among newborns discharged from facility-based KMC, evaluation of the use of a customized wrap to improve uptake of skin-to-skin practices, completion and quality of data collected on birth weight at health facilities and valuation of approaches to improve measurement of service readiness for small and sick newborns
Knowledge management	KMC is included in maternal and newborn manuals and trainings
	KMC is included in the national quality of care tool
Monitoring and evaluation	KMC services are monitored through the HMIS
	Five core KMC indicators are included in the DHIS2
Advocacy	Professional organizations advocated for the inclusion of KMC in the nurses’ curriculum.
	Professional organizations supported the development of training courses and have provided mentorship in ten district hospitals.
	One of the KMC champions has promoted KMC through mentoring three national-level paediatric and midwife mentors and has coached and supervised KMC implementation at district hospitals.
**Nigeria**	
National policy	KMC is part of newborn care policies
	KMC is part of the minimum facility-based newborn health service care package
Monitoring and evaluation	Data for KMC indicators is collected by health facilities. Summarized information in entered electronically into the DHIS2
Advocacy	KMC champions helped develop the KMC operational guidelines and played a key role in establishing KMC centers.
**Rwanda**	
National policy	KMC is part of a national policy
	KMC is part of the national health strategic plan
	KMC is included in the national guidelines for care of small babies
Country support/ implementation	KMC is provided in tertiary, teaching, provincial and district hospitals
	KMC funding mostly comes from the MOH
Monitoring and evaluation	KMC indicators are included in the HMIS. Health facilities providing KMC services track service delivery including follow-up visits using the neonatal and KMC register

KMC – Kangaroo Mother Care, HMIS – health management information system, DHIS2 - District Health Information System 2

### National policy

The MOHs in the six priority countries committed to improve the health outcomes of small babies through different strategies including the integration of KMC into policies that outline newborn care. While countries were at different stages in terms of KMC policies and guideline implementation in 2014, at the time of the last assessment, all six countries had KMC policies in place. The policies included plans for KMC scale-up including resource allocation, coverage and survival targets. For example, the Government of Bangladesh (GOB) in 2013 declared its commitment to introduce and scale up KMC at the facility level and to set a target of reducing under-five mortality to 20 per 1000 live births by 2035 [19]. While the six countries exhibited progress between the first and the last assessment, Nigeria and Ethiopia were the countries with the most progress in this dimension. In 2014, Nigeria did not have a KMC national policy in place, but by 2019, KMC had been integrated into newborn care policies making KMC part of the basic minimum package of health services. Although Ethiopia had a KMC policy drafted in 2014, in 2015, KMC was integrated into the national strategy for newborn care, as well as the health sector transformation plan.

India, Malawi and Rwanda were already ahead in terms of national guidelines at the time of the first assessment. In India, the National Guidelines for KMC and Optimal Feeding of LBW infants were released in 2014 in an effort to implement KMC at the facility level [20]. Later, in the India Newborn Action Plan (INAP), KMC was listed as a specific intervention recommended for small and sick newborns who weigh less than 2000 g [20]. The INAP highlighted the establishment of fully functional KMC unit/wards in health facilities that provide neonatal care services and identified specific coverage targets [21]. Malawi, one of the early adopters of KMC, developed national guidelines in 2005. The guidelines recommended that all babies less than 2500 g be initiated in KMC and be followed up after discharge. Through the ENAP, coverage targets were set at 75% by 2020, 80% by 2025, 85% by 2030, and 90% by 2035. In Rwanda, the MOH included KMC in the National Child Health Policy in 2009, and most recently KMC was included in the MNCH Strategic plan 2018-2024. Including KMC as part of newborn care policies has led to policy-driven actions such as setting measurable time-bound targets, developing KMC guidelines, allocating resources to train staff to provide KMC services, and designating or redesigning KMC space to advance scale-up of facility-based KMC.

### Country support/implementation

KMC services are provided at health facilities of different levels in all six priority countries, but there is variability in the coverage, type and quality of services provided and the data quality. In terms of availability of KMC services, India and Bangladesh have greatly increased the number of KMC units in the past few years. In Bangladesh, KMC is being implemented in some tertiary, teaching, and secondary/district health facilities; the number of health facilities offering KMC services increased over four times between 2017 and 2019, from 30 to 167 health facilities according to data from DHIS2 2019. In India, the Ministry of Health and Family Welfare (MOHFW) allotted funds to each state for the adaptation of KMC spaces within the special newborn care units (SNCUs). From 2016 to 2019, the number of KMC units almost tripled, growing from 265 to 782 at district hospitals. However, back in 2017, only 15% of KMC units met the MOHFW recommended number of eight beds per unit. In Nigeria, some general, tertiary and specialist hospitals, as well as some secondary health facilities provide KMC. In an assessment led by Save the Children in 31 states, 202 facilities out of 757 (27%) reported availability of KMC services [22]. However, this might be an overestimation due to different interpretations of what might constitute provision of KMC services. In Rwanda, KMC is implemented in tertiary, teaching, provincial and district hospitals, but data are needed to confirm the number of district hospitals where KMC services are functioning.

KMC coverage estimates in most countries are uncertain, as the numerator (the total of small babies who received KMC) and the denominator (the total population of small babies) are challenging to measure. However, most priority countries are starting to integrate KMC indicators into their national health management information systems (HMIS). In Malawi, it is estimated that fewer than 10% of preterm/LBW newborns receive facility-based KMC, according to data extracted from maternity reports within the District Health Information System 2 (DHIS2). All government-owned tertiary and district hospitals offer facility-based KMC services, although it is difficult to estimate whether KMC is fully operational (staff receiving in-service training on KMC, identified space for KMC and availability of functional infant scale) [18] due to inconsistent reporting through the DHIS2. In Ethiopia, an implementation study, which is expected to inform the strategy of scaling up KMC in Ethiopia, is ongoing. Coverage estimates are presented among the findings. In 2015, a study in six hospitals reported that only 14% of babies who weighed less than 2000 g were initiated in KMC, suggesting low levels of initiation [23]. KMC services are provided in facilities at different levels across the six priority countries, at the tertiary, secondary or primary level, and in some cases in teaching and private hospitals. Even though availability of KMC services has increased in the six countries in terms of number of health facilities with KMC services at different levels, coverage continues to be difficult to estimate.

### Research

Similar trends were observed in the themes selected for research over time across the six countries. Generally, by the first assessment, countries had already conducted studies or pilots about the feasibility of KMC implementation. Later, research topics about perceptions, barriers, quality of services and community KMC seemed to be of interest. During the first assessment, Nigeria had already published a study on KMC conventional care in 2004 and later conducted implementation research and studies on perceptions of KMC and attitudes of Nigerian health workers towards KMC. Malawi had conducted multiple studies on KMC service readiness and evaluation of KMC services by 2014. During the 2017 assessment, there were studies on early outcomes, customization of the wrap for skin-to-skin contact, quality of data collection on birthweight, and improving measurements of service readiness. In Bangladesh, a pilot study of KMC had been conducted at the time of the first assessment, KMC operational research was ongoing during the midline assessment, and studies on lessons learned about caring for small babies and implementation research on KMC services had already been published by the last assessment.

### Knowledge management

KMC centers of excellence are health facilities that either underwent an accreditation process through the Fundación Canguro (Kangaroo Foundation) based in Bogotá, Colombia or were considered state-of-the-art facilities, having served as training centers and model sites for KMC expansion. The centers of excellence have been building blocks in the adoption and sustainability of KMC, because in most countries, they have pioneered KMC as stand-alone facilities, served as training hubs in their respective countries, and generated evidence of practice. In Rwanda, the Muhima District Hospital was established as a center of excellence in 2007. In Malawi, the Queen Elizabeth Central Hospital and the Thyolo District Hospital are both considered KMC centers of excellence. In the late 1990s, KMC was introduced in Nigeria in the Lagos University Teaching Hospital. This and two other health facilities, the Obafemi Awolowo University Teaching Hospital (Ile-Ife), and the Federal Medical Center (Katsina) are centers of excellence. In India, five centers of excellence were established between 2003 and 2005, and they continue to be centers of excellence. While the number of centers of excellence for KMC has not increased significantly over time, these facilities continue to operate as KMC models and resource centers, contributing to the sustainability of KMC.

Integrating KMC into the curriculum of health professionals and developing KMC manuals and trainings are also important components of scaling up and sustaining KMC. In Bangladesh, KMC has been integrated into the postgraduate curriculum of physicians, as well as the curricula of nurses and midwifes. KMC training in Bangladesh is also done through trainers-of-trainers models where health providers attend trainings in other countries and train KMC teams in multiple hospitals. In India, KMC is highlighted as a strategy to be recommended to families with small babies in the handbook for community health workers (Accredited Social Health Activists, ASHAs). In Rwanda, KMC was integrated into the basic reference manual of emergency obstetrics and newborn care in 2009, into the Essential Newborn Care Reference Manual in 2011, and into maternal community health worker trainings. Continuing to embed KMC in the education process of health professionals and in training materials will facilitate the expansion of KMC.

### Monitoring and evaluation

As KMC services are offered more widely in each country, there is a need to collect, review and use KMC data in a systematic way to monitor progress, identify gaps and improve the quality of KMC services. All six priority countries have developed KMC-related indicators, although the interface for and frequency of reporting varies. Five priority countries (Malawi, Nigeria, Rwanda, Bangladesh and Ethiopia) monitor KMC services through their corresponding HMISs. India and Bangladesh achieved the most progress between the first and the last assessment. In India, in 2017, indicators were just being developed; by 2019, 782 facilities were reporting monthly on six KMC indicators through an online tracking system. In Bangladesh, in 2017, KMC indicators were being incorporated into the DHIS2 and a monthly KMC reporting form was implemented. By 2019, the DHIS2 was used to report on seven indicators related to KMC as well as ambulatory KMC follow-up data.

In some countries, the indicators have been modified to better capture the coverage of KMC services and others have been added to record follow-up visits after discharge. Nigeria and Rwanda have changed the data-reporting requirements to calculate the percentage of small babies initiated in KMC. In both countries, the denominator was recently changed to include only babies weighing 2000 g or less. There is a range in the type of KMC indicators being used to track KMC. Rwanda and Bangladesh added an indicator to track up to three KMC follow-up visits after discharge. India tracks KMC indicators through online software for SNCUs. Initially there was only one binary indicator related to KMC, but the government of India (GOI) formed working groups to develop other KMC indicators. Six indicators were developed and integrated into the SNCU online system for monthly reporting. In Bangladesh, to monitor KMC implementation, KMC indicators were incorporated into the national dashboard on DHIS2. The KMC database for DHIS2 has been tested and operationalized but not yet scaled up.

### Advocacy

KMC advocacy was assessed in two areas: professional organizations that endorse KMC and the existence of local KMC champions. Overall, there was a trend in the six priority countries for professional organizations – minimally involved in KMC advocacy initially – increasingly to support scale-up of KMC through mentoring, advocating for KMC spaces, publishing evidence-based findings and, in some cases, working at national level to include KMC in curricula, guidelines and policy documents. In Ethiopia, the Ethiopian Paediatric Society had endorsed KMC at the time of the first assessment but had taken no specific action; five years later this organization was supporting the establishment of KMC spaces, equipping them and training health workers to introduce and provide KMC services.

KMC champions, such as professional organizations members, have become instrumental in advocacy efforts. KMC champions have promoted and advocated for KMC. They also exist at different levels at the MOH, as part of the implementing partners or at health facilities. In Rwanda, KMC champions advocated for the inclusion of KMC indicators in the HMIS, increased availability of KMC units in all facilities and referral of all babies in KMC position. In Nigeria, they have supported the development of the KMC operational guidelines, and they have helped to establish KMC centers. It is evident that professional organizations and other local champions have been fundamental for KMC to become a core strategy in newborn care. However, all countries cited a lack of funding and human resources as a recurrent obstacle to expanding KMC working groups and mentoring future KMC champions.

### Priority countries compared to the countries of interest

Among the eight countries of interest, four countries (Mali, Philippines, Vietnam, and the Dominican Republic) were in similar stages of scaling up KMC as the priority countries in the strategic areas of national policy, country support and implementation, and monitoring and evaluation. China, Mozambique, and Pakistan were making progress mostly in the areas of national policy, and knowledge management, but in terms of country support and implementation, access to services was limited as KMC was implemented on a small scale. Uganda and Tanzania made some progress in the areas of country support and implementation and knowledge management. There were some facilities that offered KMC services and one or more KMC centers of excellence or state-of-the-art facilities. However, there were challenges in the other strategic areas (**Online Supplementary Document**).

## DISCUSSION

In each priority country, active engagement by the government appears to have been the major driver of progress toward scaling up KMC. Initially, governments largely resisted making specific commitments to prioritize KMC by integrating it into national newborn health policies and issuing national KMC guidelines. The priority countries received technical assistance from the KAP to devise national plans for KMC, while partners, champions and other stakeholders advanced the development of the Every Newborn Action Plan, including increasing availability of KMC services and setting coverage targets. By the last assessment, the governments of all the priority countries were actively engaged in promoting KMC, often leading the way with support of donors and partners. Local KMC champions and professional organizations stepped into key leadership roles to ensure that KMC policies, guidelines and training materials had been rolled out.

During the three assessment phases, participants consistently expressed concerns about funding gaps. As KMC centers of excellence were established and implementation research demonstrated the feasibility of scaling up KMC within existing health systems, funding needs shifted from advocacy for KMC adoption to training more health providers in KMC, increasing family and community acceptance of KMC and providing more space in facilities for KMC. While the initial assessments showed that funding for KMC was almost solely provided by donors and partners, the later assessments found that governments were increasingly contributing their own funds for sustained KMC service delivery and further scaling up. As each country takes ownership and continues to prioritize KMC, scale-up and optimal coverage seem attainable in alignment with Every Newborn milestones and targets.

The priority countries made progress in developing KMC indicators and integrating them into HMIS or other reporting systems. In most countries, monitoring of KMC through data collection and analyses has been an iterative process in which indicators have been modified and others have been added as countries have begun to appreciate the information they need to track coverage and monitor quality better. However, quantitative data during the three assessments was scarce, which prevented an accurate estimation of KMC coverage and trends over time. Now that health facilities are reporting on KMC indicators regularly, the next step is to improve the data quality while using data for decision making so that successes can be quantified.

In all priority countries there is a need to strengthen the ambulatory follow-up of small babies. This is an important component of KMC given that monitoring the health of a small baby after discharge improves early identification of danger signs, prompting proactive actions. Countries have taken different approaches to implement KMC follow-up, either by asking mothers to bring their babies to the health facility for follow-up visits after discharge or by sending community health workers to support follow-up visits at home. Ambulatory follow-up visits need to be prioritized to ensure that small babies have positive outcomes. This requires capacity building of both facility and community-based health workers, data reporting of hospital discharges and follow-up visits, and education of caregivers in the importance of complying with follow-up visits.

## CONCLUSIONS

Global commitment to work urgently towards decreasing child mortality has helped prioritize the implementation of life-saving measures such as KMC. Scaling up facility-based KMC and standardizing high-quality ambulatory follow-up after discharge will reduce morbidity and mortality among small babies. Driving factors that have facilitated the acceleration of KMC implementation in KAP priority countries have been government commitment to reduce child mortality, the technical and financial support of implementing partners, and the strong individual contributors and professional organizations who have championed KMC by driving the integration of KMC into newborn care policies and the development of KMC guidelines.

The priority countries had most notable achievements in the policy and country implementation dimensions, which resulted in making facility-based KMC a recommended intervention for the care of small babies. Integrating KMC as part of national policies for the care of newborns has laid the foundation to scale up KMC and to increase coverage. Priority countries are well on their way to accelerating the scale-up of facility-based KMC. However, as more health facilities are expected to provide KMC services, it is imperative to monitor the quality of the services and to strengthen the data-collection systems.

The number of health facilities offering KMC services increased in all the priority countries, but the percentage of small babies who are initiated in KMC remains low. To improve coverage so that KMC is fully implemented and scaled up, it is vital to address funding gaps to allocate human resources adequately, invest in research and promote acceptance of KMC. It is also important to support efforts like KAP. Through KAP, stakeholders have convened to set KMC metrics, develop national plans for KMC and learn from other countries’ experiences in KMC implementation. Continuing to support these types of initiatives will serve as a catalyst to accelerate KMC uptake and scale-up and other life-saving interventions to maximize the survival of small babies.

## Additional material

Online Supplementary Document
