# Supplementary material for: Monitoring the implementation and scale-up of a life-saving intervention for preterm and small babies: Facility-based Kangaroo Mother Care
Source: J Glob Health. 2021 Jul 24;11:14001. doi: 10.7189/jogh.11.14001 (PMC8325930; doi:10.7189/jogh.11.14001)
Supplement: Online Supplementary Document [file jogh-11-14001-s001.pdf]

Table S1

General Questions by Strategic Area

Policy KAP Metrics Reference		Questions
4. Policy KAP Metrics<br>Percentage of target countries in which national policy includes KMC as a standard of care	1a.	Is there a national policy that includes facility-based KMC as a standard of care for preterm and/or low birth weight (LBW) infants?
	1b.	If yes, when was it last updated?
	1c.	Indicate the names of each policy that includes KMC as a standard of care. Would it be possible to send a copy of the policy or let us know where it is available online?
	1d.	What are some of the gaps in these policies for KMC service provision? Is anything being done now or planned to address the gaps?
5. Policy KAP Metrics<br>Percentage of target countries with national guidelines or standards on care of preterm and low birth weight infants (aligned with WHO guidelines to include KMC)	2a.	Are there national guidelines in place about the care of preterm and LBW infants that include inpatient KMC?
	2b.	If yes, when were they last updated?<br>In your opinion, are they aligned with the 2015 WHO guidelines on care of preterm and LBW infants?<br>Would it be possible to send a copy of the guidelines or let us know where they are available online?
	2c.	If no, are there national guidelines about the care of preterm and LBW infants that include some of the components of KMC?
	2d.	To your knowledge, do guidelines cover family-centered care, and specifically, do they allow/encourage mothers/families to visit small and sick babies?

Country Support/Implementation Goal KAP Metrics		Questions
1. Country Implementation KAP Metrics<br>Percentage of target countries in which KMC is operational. Number of facilities with inpatient maternity services (aggregate of ENAP process and KMC core metric)	4a	At what levels and types of facilities is inpatient KMC implemented?<br>For each of the categories of facilities, please estimate whether facility-based KMC is implemented in (1) just one center, (2) some facilities, (3) most facilities, or (4) all facilities<br>1) Tertiary hospitals.<br>2) Teaching hospitals.<br>3) Secondary/district hospitals.<br>4) Private hospitals.<br>5) First-level health facilities with inpatient maternity.
	4b	Is there a system for tracking the availability of facility-based KMC services in your country? If yes, describe it.
	4c	Is there a system for tracking whether KMC services are operational in your country? If yes, describe it.
	4d	To the best of your knowledge, in how many facilities with inpatient maternity are KMC services operational (can you estimate the percentage of facilities)? Does this vary by level of facility?
	4e	If KMC is not offered at the national level, are there plans to scale up KMC geographically to other facilities? What partners are leading this scale up?
2. Goal KAP Metrics<br>Percentage of target countries with estimated coverage of 50% of preterm/LBW newborns initiated in facility-based KMC (aggregate of ENAP coverage and KMC core metric)	4f.	To the best of your knowledge, what percentage of preterm/LBW newborns are initiated in facility-based KMC?<br>1) < 10%.<br>2) 10-< 25%.<br>3) 25-< 50%.

Level of KMC Implementation/KAP Metrics<br>Percentage of target countries in which (1) KMC activity is limited to one center in the country, (2) KMC has spread beyond one center to a few peripheral centers in the country, (3) KMC is being implemented in a large number of public/government hospitals in the country, and (4) KMC is being implemented in the vast majority of or all public/government hospitals in the country		4) 50-<7 5%.<br>5) 75% or more.<br>6) Don't know.
	4g.	How did you arrive at that estimate (probe on sources of data)?
3. Goal KAP Metrics<br>Percentage of target countries with estimated coverage of 50% or higher of all newborns initiated in immediate skin-to-skin contact (interim indicator and proxy for skin-to-skin contact becoming routine care; can disaggregate by birth weight categories where sample size allows)	4h.	To the best of your knowledge, how routine is the practice of placing babies in skin-to-skin contact with the mother right after birth?<br>1) Very routine – practiced for all babies in all public and private facilities at all levels.<br>2) Routine practice in public facilities, but variable in private facilities.<br>3) Not routine practice in either public or private facilities.
	4i.	To the best of your knowledge, what percentage of all newborns are initiated in immediate skin-to-skin contact after delivery?<br>1) < 10%.<br>2) 10-< 25%.<br>3) 25-< 50%.<br>4) 50-< 75%.<br>5) 75% or more.<br>6) Don't know.

**Funding KAP Metrics**		**Questions**
Funding KAP Metrics<br>Percentage of countries with (1) no funding available, (2) only donor funding available, (3) combination of donor/MOH funding, and (4) no need for donor funds, fully funded by government/MOH (funds have been allocated and released)	5a.	Would you say that funding for KMC is (Please select from the following)?<br>1) There is no funding available.<br>2) There is only donor funding available.<br>3) There is a combination of donor/MOH funding.<br>4) There is no need for donor funds because KMC is fully funded by the government/MOH.
	5b.	What do you think are the most important funding gaps for KMC/care of small babies and what is being done or could be done to fill the gaps?

**Monitoring & Evaluation KAP Metrics**		**Questions**
8. M&E KAP Metrics<br>Percentage of target countries in which national HMIS includes the number of newborns who received facility-based KMC care	6a.	Are KMC indicators included in the national HMIS?
	6b.	What, if any, KMC data are recorded at health facilities? Is there a standard register/report that includes KMC? If yes, could a copy of the register and reporting forms be shared?
	6c.	What KMC indicators are currently included in the HMS? Is it possible to send a copy of the indicators?
	6d.	If no data on KMC are included in national HMIS, are there plans to include this in future? What are the main barriers? Are there any project-based KMC indicators in use?

**Knowledge Management**		**Questions**
	7a.	Are there facilities that have been nominated as centers of excellence or state-of-the-art facilities for KMC/care of preterm/LBW babies?
	7b.	If yes, how are the lessons learned at such facilities being disseminated?

	7c.	Are there KMC guidelines being developed for healthcare providers? (Skip this question if it was already addressed in the policy section.)
	7d.	Is there South-South cooperation in terms of exchanging best practices?
	7e.	Are you aware of any opportunities to exchange best practices in a global setting?

**Research**		**Questions**
	8a.	Are there any studies being conducted related to KMC in your country currently? If yes, what is the focus of the research and who is leading the research?
	8b.	To your knowledge, have any studies or program-based learning sessions been conducted in this country on the following topics?<br>• Limiting and enabling factors to appropriate uptake of KMC.<br>• Minimum dataset for KMC facility-based inputs and outcomes.<br>• Barriers and enablers for families continuing KMC post-discharge.<br>• Family compliance with continuing KMC at home following discharge.<br>• Follow-up care for babies discharged on KMC from hospital.<br>• Coverage indicators for KMC.<br>• Effectiveness of healthcare providers in training mothers and facilitating KMC.<br>• Minimum number of hours per day of skin-to-skin contact needed to obtain the benefits associated with KMC.<br>• Establishing tools and processes for referral care.<br>• KMC counselling during ANC.

**Advocacy KAP Metrics**		**Questions**
7. Advocacy KAP Metrics<br>Percentage of target countries with endorsements for KMC from national professional bodies and/or flagship teaching hospitals with operational KMC<br>*Probe for standards taught in certain specialties such as paediatrics or training programs.*	9a.	Are there professional organizations that endorse KMC?
	9b.	If yes, please list the organizations and indicate when they made the endorsements (if possible).
	9c.	How are these organizations engaged in scaling up KMC?
	9d	Is KMC taught as a standard of care in teaching hospitals in your country? If not, what are the reasons/barriers?
	9e.	How is KMC typically taught (i.e., textbook, lecture, on the ward demonstration, etc.)?
	9f.	Has KMC been taught/discussed in any paediatric meetings/conferences?
Champions KAP Metrics<br>Percentage of countries with (1) no local champion, (2) local champion(s) who have limited time and resources to devote to KMC, limited track record(s) in promoting KMC and limited national presence, (3) local champion(s) with track record(s) of promoting KMC OR national presence, but with limited time and resources to devote to KMC, and (4) strong local champion(s) with track record(s) of KMC promotion, significant time and resources to devote to KMC and strong presence at a national level	9g.	When thinking about the champions who promote KMC in this country. Would you say?<br>1) There are no local champions.<br>2) Local champion(s) exist, but with limited time and resources to devote to KMC, limited track record(s) in promoting KMC and limited national presence.<br>3) Local champion(s) with track record(s) of promoting KMC OR national presence, but with limited time and resources to devote to KMC.<br>4) Strong local champion(s) with track record(s) of KMC promotion, significant time and resources to devote to KMC, and strong presence at a national level.
	9h.	Can you provide more information on KMC champions in your country? Are the champions clinical practitioners, public health practitioners or a mix of both?

	9i.	What have been some successes of champions around KMC since 2014? What are some of the challenges they have faced?

**Other Questions (Tailored to Each Country)**		**Questions**
Community engagement and social behaviour change communications (SBCC)	10a.	Are there any national or subnational SBCC campaigns or efforts that address care of small/preterm babies, including KMC? If yes, describe.
	10b.	Are there community health workers for maternal and newborn health at community level who are trained to support raising awareness around KMC and helping to follow up babies on KMC? If yes, describe.
General progress on KMC over time	10c.	In your opinion, what has been the most significant change in terms of availability and quality of KMC services in your country in the last 3 years (since 2014)? Why?
Future actions	10d.	What do you think is the most important step to improve coverage of quality KMC in the next 3 years? Who needs to do what to make that happen?
Perspectives on the KMC Acceleration Partnership (KAP)	10e.	What do you think has been the most important contribution of the KAP to accelerating KMC in your country?
	10f.	What do you think is the most important action the KAP could take to support your country to accelerate KMC in the next 3 years?
Key Stakeholders	10g.	Please name up to five people/organizations you think are critical to engage to scale up KMC in your country.

Table S2

Policy

**Annex 2. KAP Metrics Reference**		**Questions 2019 Assessment**
4. Policy KAP Metrics<br><br>Percentage of target countries in which national policy includes KMC as standard of care<br><br>*For those countries that scored 4 in 2014 we may want to reference that but get specific information (question1c) and ask if they could send it to us – as the 2014 questions combined both policy and guidelines, so it was a bit unclear.*	1a.	Is there a national policy that includes facility-based KMC as a standard of care for preterm and/or LBW infants?
	1b.	If yes, when it was last updated?
	1c.	Indicate the names of each policy that includes KMC as a standard of care. Would it be possible to send a copy of the policy or let us know where it is available online?
	1d.	What are some of the gaps in these policies for KMC service provision? Is anything being done now or planned to address the gaps?
	1di.	What have been some of the advances in the past two years on the scale-up of KMC?
5. Policy KAP Metrics<br><br>Percentage of target countries with national guidelines or standards on care of preterm and low birth weight infants (aligned with WHO guidelines to include KMC)	2a.	Are there national guidelines in place about the care of preterm and LBW infants that include inpatient KMC?
	2b.	If yes, when were those last updated?<br><br>In your opinion, are they aligned with WHO 2015 guidelines on care of preterm and LBW infants?<br><br>Would it be possible to send a copy of the guidelines or let us know where it is available online?
	2c.	If no, are there national guidelines about the care of preterm and LBW infants that include some of the components of KMC?
	2d.	To your knowledge, do guidelines cover family-centred care, and specifically do they allow/encourage mothers/families to visit with small and sick babies?
	2e.	Please clarify if the ambulatory follow-up of small babies who are enrolled in KMC, is specified in the national guidelines. **Has there been any progress increasing follow-up visits?**
	2f.	**Has there been progress in scaling up KMC in the past two years?**

Country support/implementation

**Goal KAP Metrics**		**Questions**

1.Country Implementation KAP Metrics	4a	At what levels and types of facilities is inpatient KMC implemented? For each of the categories of facilities, I would like you to estimate whether facility-based KMC is implemented in 1) just one centre; 2) some facilities; 3) most facilities; or 4) all facilities<br><br>1) Tertiary hospitals<br>2) Teaching hospitals<br>3) Secondary/district hospitals<br>4) Private hospitals<br>5) First level health facilities with inpatient maternity
	4b	Is there a system for tracking availability of facility-based KMC services in your country? If yes, describe
	4c	Is there a system for tracking whether KMC services are "operational" in your country? If yes, describe
	4d	To the best of your knowledge, in how many facilities with inpatient maternity are KMC services operational? (Can you estimate percentage of facilities). Does this vary by level of facility?
	4e	If KMC is not offered at the national level, are there plans to geographically scale up KMC to other facilities? What partners are leading this scale up?
2. Goal KAP Metrics	4f.	To the best of your knowledge, what percentage of pre-term/LBW newborns are initiated on facility-based KMC?<br>1) <10%<br>2) 10-<25%<br>3) 25-<50%<br>4) 50-<75%<br>5) 75% or more<br>6) Don't know
	4g.	How did you arrive to that estimate? (Probe on sources of data)
Goal KAP Metrics<br><br>Percentage of target countries with estimated coverage of 50% or higher of all newborns initiated on immediate skin-to-skin (interim indicator and proxy for skin-to-skin becoming routine care; can disaggregate by birth weight categories where sample size allows)	4hi.	Is skin-to-skin included as part of the KMC guidelines?
	4h.	To the best of your knowledge, how routine is the practice of placing babies in skin-to-skin position with the mother right after birth:<br>1) Very routine – practiced for all babies in all public and private facilities at all levels<br>2) Routine practice in public facilities, but variable in private facilities<br>3) Not routine practice in either public or private facilities
	4i.	To the best of your knowledge, what percentage of all newborns are initiated on immediate skin-to-skin after delivery?<br>1) <10%<br>2) 10-<25%<br>3) 25-<50%<br>4) 50-<75%<br>5) 75% or more<br>6) Don't know

Funding KAP Metrics		Questions
Funding KAP Metrics	5a.	Would you say that funding for KMC is (Please select from the following):<br>1) There is no funding available<br>2) There is only donor funding available<br>3) There is a combination of donor/MOH funding<br>4) There is no need for donor funds because KMC is fully funded by the government/MOH.
	5b.	What do you think are the most important funding gaps for KMC/care of small babies and what is being done or could be done to fill the gaps?

Monitoring & Evaluation

M&E KAP Metrics		Questions
8. M&E KAP Metrics	6a.	Are there KMC indicators included in the national HMIS?
	6b.	What, if any, KMC data are recorded at health facilities? Is there a standard register/report that includes KMC? If yes, could a copy of the register and reporting forms be shared?
Percentage of target countries in which national HMIS includes the number of newborns who received facility-based KMC care	6c.	What KMC indicators are currently included in the HMS? Is it possible to send a copy of the indicators?
	6d.	If no data on KMC are included in national HMIS, are there plans to include this in future? What are the main barriers? Are there any project-based KMC indicators being used?
	6e.	What, if any, KMC ambulatory follow-up data are recorded at health facilities? Is there a standard register/report that includes KMC ambulatory follow-ups? If yes, could a copy of the register and reporting forms be shared?

Knowledge Management

N/A Knowledge Management		Questions
	7a.	Are there facilities that have been nominated as centers of excellence or state of the art facilities for KMC/care of preterm/LBW babies?
	7b.	If yes, how are the lessons learned at such facilities being disseminated?
	7c.	Are there KMC guidelines being developed for healthcare providers?
	7d.	Is there South-South cooperation in terms of exchanging best practices?

	7e.	Are you aware of any opportunities to exchange best practices in a global setting?
	7f.	Are you aware of any opportunities to exchange best KMC practices at the national level? How about exchanging best practices?

Research

**N/A Research**		**Questions**
	8a.	Are there any studies being conducted related to KMC in your country currently? If yes, what is the focus of the research and who is leading the research?
Probe for other type of studies such as program-based, not only formal studies.	8b.	To your knowledge, have any studies or program-based learning been conducted in this country on the following topics:<br>· Limiting and enabling factors to appropriate uptake of KMC<br>· Minimum dataset for KMC facility-based inputs and outcomes<br>· Barriers and enablers for families continuing KMC post-discharge<br>· Family compliance with continuing KMC at home following discharge<br>· Follow-up care for babies discharged on KMC from hospital<br>· Coverage indicators for KMC<br>· Effectiveness of health care providers in training mothers and facilitating KMC<br>· Minimum number of hours per day of skin-to-skin care needed to obtain the benefits associated with KMC<br>· Establishing tools and processes for referral care<br>· KMC counseling during ANC

Advocacy

**Advocacy KAP Metrics**		**Questions**
7. Advocacy KAP Metrics	9a.	Are there professional organizations that endorse KMC?
	9b.	If yes, please list the organizations and indicate when they made the endorsements (if possible)
Percentage of target countries with endorsement for KMC from national professional bodies and/or flagship teaching hospital with operational KMC	9c.	How are these organizations engaged in scaling up KMC?

	9d	Is KMC taught as standard of care in teaching hospitals in your country? If not, what are the reasons/barriers?
Probe for standards taught in certain specialties such as pediatrics or training programs.	9e.	How is KMC typically taught? (i.e., textbook, lecture, on the ward demonstration, etc.)
	9f.	Has KMC been a sessions/discussed in any pediatric meetings/conferences?
Champions KAP Metrics<br><br>Percentage of countries with: 1) no local champion; 2) Local champion(s) exist, but with limited time and resources to devote to KMC, limited track record in promoting KMC, and limited national presence; 3) Local champion(s) with track record of promoting KMC OR national presence, but with limited time and resources to devote to KMC; and 4) Strong local champion(s) with track record of KMC promotion, significant time and resources to devote to KMC, and strong presence at a national level	9g.	When thinking about the champions who promote KMC in this country. Would you say:<br><br>1) There are no local champions<br>2) 2) Local champion(s) exist, but with limited time and resources to devote to KMC, limited track record in promoting KMC, and limited national presence<br>3) Local champion(s) with track record of promoting KMC OR national presence, but with limited time and resources to devote to KMC<br>4) Strong local champion(s) with track record of KMC promotion, significant time and resources to devote to KMC, and strong presence at a national level
	9h.	Can you provide more information on KMC champions in your country? Are the champions clinical practitioners or public health practitioners, or a mix of both?
	9i.	What have been some successes of champions around KMC since 2014? What are some of the challenges they have faces?

Other

**Community engagement and Social Behavior Change Communications**		**Questions**
Community engagement and Social Behavior Change Communications	10a.	Are there any national or subnational SBCC campaigns or efforts that address care of small/preterm babies, including KMC? If yes, describe.

	10b.	Are there CHWs for maternal and newborn health at community level who are trained to support raising awareness around KMC and helping follow-up babies on KMC? If yes, describe.
**General progress on KMC over time**	10c.	In your opinion, what has been the most significant change in terms of availability and quality of KMC services in your country in the last 3 years (since 2014)? Why?
**Future actions**	10d.	What do you think is the most important action that needs to be taken to improve coverage of quality KMC in the next 3 years? Who needs to do what to make that happen?
**Perspectives on the KMC Acceleration Partnership**	10e.	What do you think has been the most important contribution of the KAP to accelerating KMC in your country?
	10f.	What do you think is the most important action the KAP could take to support your country to accelerate KMC in the next 3 years?
**Key Stakeholders**	10g.	Please name up to 5 people/organizations you think are critical to engage to scale-up KMC in your country.

Table S3

Country Profiles of six priority countries and China

Country profiles for six priority countries (Bangladesh, Ethiopia, India, Malawi, Nigeria and Rwanda) and one country of interest (China) were created to present the information collected during the 2014, 2017, and 2019 assessments. China provided information in the 2017 and 2019 assessments.

KANGAROO MOTHER CARE IN BANGLADESH

OVERVIEW

The experience of facility-based kangaroo mother care (KMC) in Bangladesh is relatively recent, though a few facilities have been providing KMC services for over a decade. In 2013, the Government of Bangladesh signed onto A Promise Renewed (APR) as a sign of its determination and commitment to reduce child deaths to 20 per 1,000 live births by 2035. Through this commitment, KMC has been integrated into newborn care for preterm/low-birthweight (LBW) babies in an effort to reduce preventable newborn deaths. The implementation of KMC has increased in the past few years. In 2019, KMC was practiced in 167 health facilities compared to 30 in 2017 (data form DHIS2 2019). The government's leadership—including the Directorate General of Health Services (DGHS) and the Directorate General of Family Planning (DGFP)—and the support of implementers, professionals, and donors has been instrumental in achieving key milestones for scaling up KMC services throughout the country.

Table 1. Status of KMC in Bangladesh by strategic area

Domain	Prior to and During 2014	2015-2017	2017-2019
**Policy**			
National Health Policy	In 2013, the National Core Committee of Neonatal Health (NCC-NH) under the Ministry of Health and Family Welfare (MOHFW) adopted a policy that integrated KMC as part of newborn and maternal health. The Government of Bangladesh (GOB) in 2013 declared its commitment through APR to introduce and scale-up KMC at the facility level with continuation at home (NCC-NH, Minutes of the 6th meeting 2013). In	• In 2015, the Bangladesh Every Newborn Action Plan (BENAP) was instituted, with benchmarks aligned with the APR. The GOB, UNICEF, World Health Organization (WHO), US Agency for International Development (USAID), Saving Newborn Lives (SNL), MaMoni Health Systems Strengthening (HSS), International Center for Diarrhoeal Disease Research, Bangladesh (ICDDR,B) and other partners were involved in this process. There are	• The GOB re-established a National newborn health program (NNHP) in 2017.<br>• The national newborn strategy, which includes KMC, is under revision.<br>• KMC services have been established in 167 health facilities (data from DHIS2), reaching 62% of the target set by the MOHFW in the HPNSDP of establishing KMC services in 270 facilities by June 2022.

	a renewed declaration, the GOB reiterated its commitment for ending preventable child deaths by 2035 in the APR. The target to reduce under-five mortality was set to 20 per 1,000 live births by 2035 by implementing different strategies, including KMC, for preterm/LBW babies.[1,2]	two main objectives in the BENAP that focus on KMC: the establishment of counselling on KMC practice at facilities, including the provision of follow-up KMC services using community health workers (CHWs), and the establishment of centers of excellence for KMC in tertiary- and secondary-level facilities.[3]<br>• In the APR, the targets for KMC initiation were set at 20% for public health facilities at the Upazila Health Complexes (UCH; sub-district) and above by 2016, and at 50% for public health facilities at the UHCs and above to provide KMC services by 2020.<br>• In 2016, KMC was included as an essential service for preterm/LBW newborns at UHCs, district hospitals (DHs), and maternal and child welfare centers (MCWCs).[4]<br>• KMC is included as a priority of newborn health intervention in the Program Implementation Plan of the Health, Population, and Nutrition Sector Program (HPNSDP) of MOHFW.	
National Guidelines	A technical sub-group (TSG) was formed—the National Technical Working Committee on Newborn Health (NTWC-NH), in 2013 to develop a KMC guideline and protocols. The KMC National Guideline was finalised in 2014. The main objective of the guideline is to introduce, expand and strengthen	• The KMC national guideline was approved in 2015.<br>• The KMC training manual was developed in December 2015 by KMC TSG.<br>• A KMC counselling material, job aid (booklet), was developed in 2016 with technical support from SNL. The booklet includes basic information on	• The KMC national guideline has been fully adopted and rolled out.

	KMC practices and effective national scale-up. The guideline is in line with the (WHO) KMC guidelines. The guideline is for healthcare providers and managers.	KMC, position, feeding, monitoring, follow-up, danger signs, etc. It is in the approval process.	
**Country Support/Implementation**			
Levels and types of facilities implementing KMC	• LAMB Hospital in Parbatipur, in Dinajpur, has been implementing KMC since 1999.<br>• The ICDDR,B Matlab, a rural health facility, started providing KMC services in 2007 for babies weighing less than 2,000 g as part of the Maternal, Neonatal and Child Health (MNCH) project[5] for research purposes.	• In the Kushtia District, the SNL program supports the MOHFW to implement the Comprehensive Newborn Care Package (CNCP), inclusive of KMC, to demonstrate a newborn care package through the public health system.[4] The MOHFW adopted the package, which will be scaled up throughout the country through the National Newborn Health Program (NNHP, 2017-2022). SNL provided technical support for the operational plan of the NNHP, allocated the budget, made logistic and training plans, and incorporated an operational plan-level indicator. The target coverage for KMC by 2020 was set to 100 facilities[6] and at the 70 district-level MCWCs under the Directorate General of Family Planning (DGFP).<br>• MaMoni HSS is supporting the MOHFW to introduce and scale up priority newborn interventions in six districts in both DGHS and DGFP facilities. In addition, national-level technical assistance is provided to scale up interventions at the national scale. MaMoni has been supporting the implementation of KMC in 15 district-/upazila-level facilities.	• A total of 167 KMC units have been established (data from DHIS2 2019)<br>• KMC is being implemented in some tertiary-, teaching-, secondary/district-health facilities, and in one private hospital.

		• UNICEF is supporting KMC at national-level hospitals and in districts.<br>• Overall KMC is practiced in 30 facilities (as of June 2017), which include some sub-district level hospitals and medical college hospitals.<br>• According to the *National Newborn Health Bulletin* in 2016, KMC was introduced as a pilot in 21 primary-, secondary-, and tertiary-level DGHS and DGFP facilities of Kushtia, Noakhali, Habiiganj, Jhalokati, Lakshmipur, and Dhaka, where space was designated for KMC services, providers were trained, a recordkeeping and reporting system was established, and managers and CHWs were sensitised.[6]	
Percentage of LBW newborns initiated in facility-based KMC		• In 2017, 2.13% of babies born < 2,000 g were initiated in KMC services (data from DHIS2, 2017)<br>• The emergency obstetric and newborn care (EmONC) and the KMC monthly reporting were linked and the information about the number of LBW babies under 2,000 g (derived from EmONC) and the initiation of KMC was incorporated into the health management information system (DHIS2). However, this is not fully functional yet. By the 4th HPNSP, this will be scaled up nationally to be able to obtain national data. Currently, this data is provided by KMC facilities, but	• In 2019, 19.10% of babies born < 2,000 g were initiated in KMC services (data from DHIS2 2019).<br>• In 2018, 8.07% of babies born < 2,000 g were initiated in KMC services (data from DHIS2 2018).<br>• All facilities providing KMC services do monthly reporting through the DHIS2. However, data quality can be improved.

		data at the national level are not available.	
Funding		Government funding for KMC and newborn care will increase in the upcoming years. There have been costed plans and funding allocations for KMC activities. It is the first time that the government has adopted a new newborn program, the National Newborn Health Program (NNHP), which includes KMC. Along with GOB funding for KMC, donors also provide funding.	There is a separate budget in the operation plans of MOHFW for training and supplies for the establishment of KMC spaces. The GOB set the target of establishing 270 KMC spaces by 2022. Currently, funds for KMC are a combination of donor and MOH funding.
**Research**			
Major or program-based studies currently being conducted related to KMC	There was a pilot conducted in Gopalgonj district to study the use of KMC in rural communities.[7]	A study was conducted about implementing KMC in a resource-limited setting in rural Bangladesh[5] and challenges of implementation of KMC.[8] There is also KMC operational research being conducted by ICDDR,B with the support of SNL. The baseline was completed in 2015 and the end line should be completed in 2017. The tentative date for disseminating the results was November 2017.	• BRAC University led a study on "Implementation research on KMC services in Bangladesh" in eight health facilities. Authors found that factors that played a key role in the successful implementation of KMC were service providers who are motivated and dedicated, the presence of KMC champion(s), and implementing partners who provide technical assistance. Barriers to delivery of KMC services were limited skilled human resources staff and heavy workload. The study concluded that KMC should be included in the medical and nursing curriculum to further develop health professionals' skills.[9]<br>• MaMoni-MNCSP initiated a study "Generating lessons on strengthening small and sick newborn management including post discharge follow up in MaMoni MNCSP district of

			Bangladesh". The dissemination of results is planned for 2021.
**Knowledge Management**			
Centers of excellence or state-of-the-art facilities for KMC/care of LBW babies	Since 2013, WHO has facilitated demonstrations of the use of KMC in Dhaka Shishu Hospital, the largest tertiary-level children's hospital in Bangladesh to improve the survival of premature and LBW newborns.	• WHO provided financial support to establish the first national-level training center on KMC in Dhaka Shishu Hospital.<br>• There is an SNL-led learning lab in the Kushtia district. Among the six facilities in Kushtia, one primary level facility (UHC Kumarkhali) provides good KMC services in respect of case load, adherence, positioning, duration and follow up.<br>• SNL provides technical and logistic support to train doctors and nurses on KMC at two facilities of the DGFP. These facilities will be used as training centers by the end of 2017.	
KMC manuals, trainings and campaigns		• KMC was incorporated into the postgraduate curriculum of physicians, but not as a programmatic approach. KMC has not been incorporated into undergraduate curriculums.<br>• KMC was incorporated in the nurses', midwifes' and family welfare visitors' curriculums in 2017. The inclusion of KMC in the Family Welfare Assistants (FWA) curriculum is in process. Ideally, the classroom learning will be coupled with demonstration trainings for health providers to hone their KMC skills.<br>• UNICEF supported one specialised hospital (Bangabandhu Sheikh Mujib	• The prematurity info pack was developed, which includes a KMC booklet, KMC pocketbook, KMC brochures for service providers for CHWs and for mothers and families, KMC tool kit for service providers, KMC infographic, KMC flip chart, KMC posters and stickers, and KMC follow-up card.

		Medical University, or BSMMU) to ensure KMC training of service providers.[10]<br>• In 2014, a national master trainer's pool was formed including four neonatologists, one gynaecologist and SNL staff to attend a training program in two facilities (KEM Hospital and D Y Patil Hospital) in Mumbai, India. They contributed with the development of the guidelines and the training of trainers at the national level.<br>• According to UNICEF's Annual Progress Report, five doctors and two nurses attended a training of trainers on KMC at the All India Institute of Medical Science to build their capacity before establishing a national training center on KMC at BSMMU. They will train KMC teams (three doctors and five nurses per team) in 15 selected hospitals in 14 districts.[11]<br>• Under the leadership of professionals headed by Professor M. A Mannan, a KMC reference group was formed where service providers can seek the experts' opinions regarding KMC services. Also, professionals will conduct site visits to sub-district level KMC facilities to exchange knowledge and share lessons learned about KMC.	

**Monitoring and Evaluation**			
KMC indicators included in the national HMIS		A monthly KMC reporting form was developed for incorporation into DHIS2. KMC indicators are being incorporated into the national NBH dashboard on DHIS2.<br>The KMC database was finalised in November 2016 for the DHIS2 and was endorsed by the DGHS. The KMC database for DHIS2 was tested in Kushtia in January 2017. The database was operationalised for that DGHS facility but not scaled up. DGFP is still maintaining the paper base reporting system. Initiatives are being taken to make it functional.	• Tracking of KMC services is being piloted in urban areas.<br>• KMC related indicators included in the DHIS2:<br>- Number of live births.<br>- Number of babies born < 2,000 g.<br>- Number of babies who received KMC.<br>- Number of babies born in facilities who received KMC.<br>- Number discharged after KMC as per protocol.<br>- Number of babies discharged after KMC on request. Number of babies who died during KMC.<br>- Number of babies who received 1st, 2nd, 3rd, and/or 4th follow-up.
KMC data recorded at health facilities		Only facilities that provide KMC services report on the DGHS using the KMC Monthly Report Form.	Ambulatory KMC follow-up data is recorded in the KMC follow-up card and the KMC register, and it is reported through the DHIS2.
**Advocacy**			
Professional organizations that endorse KMC	The National Technical Working Committee on Newborn Health (NTWC-NH), which is part of the MOHFW, endorsed KMC for national scale-up in July 2013 and it formed a technical sub-group on KMC for the development of KMC guidelines and protocols.[12] NTWC and TSG are led by professionals with managers from government, development partners, international NGOs, and professional societies and experts.	The Bangladesh Paediatric Association, the Obstetrical and Gynaecological Society of Bangladesh, the Bangladesh Neonatal Forum, and the Bangladesh Perinatal Society, the Bangladesh Nurses Association, and the Bangladesh Midwifery Society endorsed a policy statement for universal use of KMC. These professional organizations, which work for health services are committed and engaged with the scale-up of KMC by promoting the integration of KMC services in neonatal care and for the KMC indicators to be	

		included in the health management information system.	
Awareness campaigns		• CHWs are being oriented to raise awareness about KMC referral to health facilities and follow-up after discharge. They are trained to observe the duration of KMC and to address barriers in places that provide fully functional KMC services (30 facilities).<br>• The USAID-funded social and behaviour change communication project, called Ujjiban, is supporting the MOHFW to develop newborn health mass media communication materials. MaMoni, HSS and SNL will support the development of messages for the campaign.<br>• Information Education and Motivation Unite and DGFP are also planning to develop a newborn campaign that includes KMC.	• A total of 700 front-line health workers in ten MaMoni MNCSP districts have been trained on CNCP, which includes KMC. CHWs have a one day orientation package on KMC referral and follow-up including utilisation of the community follow-up register. However, this has not been scaled up yet.<br>• Videos on KMC have been developed as well as awareness activities at the community level in urban areas. They include puppet plays about KMC.
Champions	Professor Azad Chowdhury of Dhaka Shishu Hospital, a private hospital, initiated a KMC corner in the hospital.	• Professor Mohammad Shahidullah, chair of the NTWC-NH, president of the Bangladesh Medical and Dental Council and the Bangladesh Perinatal Society, and Professor at BSMMU, acts as a key player influencing policymakers to incorporate KMC.<br>• Professor Chowdhury, director and head of neonatology at Dhaka Shishu Hospital and secretary general of the Bangladesh Paediatric Association, is the chair of the TSG on KMC who plays a vital role in KMC guideline development, training manual	• Professor Mohammad Shahidullah, Professor Chowdhury, Professor Mannan, Professor Mahbubul Haque and Dr.Khaleda Islam all continue advocating for KMC.

		development, and initiation of KMC at tertiary-level hospitals.<br>• Professor Mannan, chair of the neonatology department at BSMMU and secretary general for the Bangladesh Neonatal Forum, is involved in KMC guideline development, establishment of KMC services in BSMMU, and is a key member of the of National KMC reference group.<br>• Professor Mahbubul Haque, professor of neonatology at Dhaka Shishu Hospital, plays a vital role in the operationalisation of KMC trainings at the national level.<br>• Dr.Khaleda Islam, director of primary healthcare and program manager of Integrated Management of Childhood Illnesses and the NNHP at DGHS, and Dr.Farid Uddin Ahmed, deputy director (services) and program manager for newborn and child health for DGFP, who are responsible for newborn health program implementation under new NNHP, will be the champions, as they have the mandate to scale up KMC nationally.	

Table 2. Demographic and Health Survey (DHS) proxy indicators for kangaroo mother care

DHS Indicators Related to KMC		Bangladesh DHS, 2014	Bangladesh DHS, 2017-2018[13]
**Identification of LBW Babies**	**Characteristic**	**Percentage**	**Percentage**
Percentage distribution of live births in the 3 years preceding the survey by mother's estimate of baby's size at birth, according to background characteristics	*Very small*	6.8	N/A
	Smaller than average	13.2	N/A
Percentage of births that have a reported birthweight		N/A	N/A
Percentage of babies weighing less than 2.5 kg among births with a reported birthweight		N/A	N/A
**Initial Breastfeeding Indicators**		**Percentage**	**Percentage**
Percentage of children born in the 2 years preceding the survey who started breastfeeding within 1 hour of birth		50.8	68.7
Percentage of children born in the 2 years preceding the survey who started breastfeeding within 1 day of birth		89.1	N/A
**Skin-to-Skin Contact Indicator**		**Percentage**	**Percentage**
Percentage of births that had skin-to-skin contact for the most recent live birth in the 3 years preceding the survey		24.7	N/A

CHALLENGES

- The need for individual KMC case tracking and the operationalisation of monitoring and evaluation tools is apparent.
- Low motivation and mentorship of health providers to provide KMC are key factors that might be contributing to the slow uptake of KMC.

LESSONS LEARNED

- Government commitment and a positive policy environment is crucial for the acceleration and sustainability of KMC services. The commitment of the MOHFW, which started in 2013, has resulted in achievements such as the development of KMC guidelines, manuals, monitoring tools and the integration of KMC into the NNHP.
- The joint efforts of members of the National Technical Working Committee for Newborn Health led to the decision and commitment to scale up KMC in Bangladesh.
- The identification of champions at the national level has accelerated the progress of KMC activities in Bangladesh, from the establishment of KMC facilities to participating in committees to scale up KMC.
- The engagement of partners supporting the DGHS and DGFP in the capacity-building of service providers and facility readiness has proved essential for the acceleration of KMC.
- Motivation and ownership of KMC from managers and providers has been crucial to ensure that KMC services are sustainable.

FUTURE ACTIONS

- Disseminate the findings of the Saving Newborn Lives program in the Kushtia district.
- Identify local-level champions who will motivate and mentor new champions.
- Develop and implement of a tracking system for KMC services.
- Establish KMC learning platform among MOHFW, professionals, donors, United Nations, development partners, and nongovernmental organizations.

DOCUMENTS AND RESOURCES

Document Title	Link to Document
KMC information brochure for mothers from Bangladesh (2013)	https://www.healthynewbornnetwork.org/hnn-content/uploads/KMC-brochure-for-mothers-Bangladesh.pdf
Television report about kangaroo mother care in Matlab facility	http://www.rockhopper.tv/films/detail/kangaroo-mother-care-in-bangladesh
Newborn care practices in rural Bangladesh: Implications for the adaptation of kangaroo mother care for community-based interventions (2014)	https://www.sciencedirect.com/science/article/pii/S0277953614006522
Daily newspaper report about kangaroo mother care in Bangladesh	https://www.healthynewbornnetwork.org/news-item/kangaroo-mother-care-helps-ensure-health-risk-newborns/
Implementing kangaroo mother care in a resource-limited setting in rural Bangladesh (2015)	https://doi.org/10.1111/apa.12929
Challenges of implementation of kangaroo mother care (KMC) in selected public health facilities of Bangladesh (2016)	http://fundacioncanguro.co/wp-content/uploads/2018/01/44_rezaul_hasan_poster-1.pdf
WHO organizes training on kangaroo mother care	http://www.searo.who.int/bangladesh/enbankmcworkshop/en/
Assessment of health facility capacity to provide newborn care in Bangladesh, Haiti, Malawi, Senegal, and Tanzania (2017)	https://www.ncbi.nlm.nih.gov/pmc/articles/PMC5804038/

REFERENCES

1. Bangladesh DGHS. *Bangladesh: National Newborn Health Situation Analysis Report 2014*. Dhaka: Bangladesh DGHS; c2014.

2. Bangladesh DGHS. *National Guideline on Kangaroo Mother Care 2014*. Dhaka: Bangladesh DGHS; c2014.

3. Bangladesh Ministry of Health and Family Welfare (MOHFW). *Bangladesh Every Newborn Action Plan*. Dhaka: Bangladesh MOHFW; c2015.

4. Bangladesh MOHFW. *Bangladesh Policy Statement for Universal Use of Kangaroo Mother Care for Preterm and Low Birthweight Infants Joint Statement.* Dhaka: Bangladesh MOHFW; c2016.

5. Pervin J, Gustafsson FE, Moran AC, Roy S, Persson LA, Rahman A. Implementing kangaroo mother care in a resource-limited setting in rural Bangladesh. *Acta Paediatr*. 2015;104(5):458–465. doi:10.1111/apa.12929.

6. Bangladesh DGHS. *National Newborn Health Bulletin*. Dhaka: Bangladesh DGHS; c2016.

7. Hunter EC, Callaghan-Koru JA, Mahmud AA, et al. Newborn care practices in rural Bangladesh: implications for the adaptation of kangaroo mother care for community-based interventions. *Soc Sci Med*. 2014;122:21–30. doi:10.1016/j.socscimed.2014.10.006.

8. Siraj W, Rubayet S, Hossain A, Islam N, Ahsan Z, Hasan R. *Challenges of Implementation of Kangaroo Mother Care (KMC) in Selected Public Health Facilities of Bangladesh*. Dhaka: Save the Children; c2016.

9. Azad M, Azad K. *Plenary: Implementation Research on Kangaroo Mother Care Services in Bangladesh*. Dhaka: Bangladesh Directorate General of Health Services (DGHS); c2019.

10. World Health Organization (WHO). WHO organizes training on kangaroo mother care. Geneva: WHO; c2017.

11. UNICEF. *UNICEF Annual Report 2015 Bangladesh*. New York: UNICEF; c2015.

12. Bangladesh Neonatal Forum (BNF). Newborns in Bangladesh: where we need to focus on. Presented at: BNF Conference 2016; November 29; Krishibid Institution, Khamar Bari, Dhaka, Bangladesh; c2015.

13. Bangladesh DHS. *Bangladesh Demographic and Health Survey 2017–18 Key Indicators*. Dhaka: Bangladesh DHS; c2017–18.

KANGAROO MOTHER CARE IN NIGERIA

OVERVIEW

In Nigeria, KMC as a method for caring for small babies has been systematically reintroduced through the US Agency for International Development's ACCESS program in 2007. Since then, there have been efforts to integrate KMC as part of the standards for newborn care. The commitment of the Federal Ministry of Health (FMOH) to increase KMC coverage has been noticeable. In 2008, with the help of partners, the FMOH adapted a KMC Training Manual, and in 2013, it included two KMC indicators in the health management information system. The FMOH launched the Nigeria Every Newborn Action Plan, which included KMC as part of the essential care for preterm/low birth weight (LBW) babies in 2016. Most recently, the FMOH included KMC in the Reproductive, Maternal, Newborn, Child, Adolescent Health Plus Nutrition Agenda 2017-2030[1] and in the National Strategic Health Development Plan II 2018 -2022.[2] Also, with partners' support, the FMOH finalised the guidelines for KMC.

The FMOH has been committed to situating facility-based KMC services within the wider context of newborn health. As KMC is included in health policies, there is a need for behaviour change campaigns, displays of KMC posters in healthcare facilities and awareness activities to engage healthcare providers and mothers. There is also a need for data quality improvement, data use and dissemination of findings, which could help to make KMC a widely practiced strategy in Nigeria.

Table 1. Status of KMC in Nigeria by strategic area

Domain	Prior to and during 2014	2015-2017	2017-2019
**Policy**			
National Health Policy	There was no national KMC policy in Nigeria, but KMC was incorporated in the Integrated Maternal Newborn, and Child Health strategy of the FMOH in 2007.[3] KMC was also included in the integrated Facility-Based Newborn Care package developed by the FMOH in 2014.[4]	• The Nigeria Every Newborn Action Plan (NiENAP), which covers KMC, was launched in 2016 by the FMOH. KMC was included in the NiENAP with coverage targets of 65% by 2020, 85% by 2025, and 95% by 2030.[5]	KMC was included in the Reproductive, Maternal, Newborn, Child, Adolescent Health Plus Nutrition agenda 2017-2030 (RMNCAH+N) strategy/policy document under the Basic Minimum Package of Health Services (BMPHS)[1] and in the National Strategic Health Development Plan II (NSHDPII) 2018 -2022 as part of the newborn health package.[2]
National Guidelines	• The FMOH included KMC in the Infant and Young Child Feeding Guidelines. KMC was covered in a newborn situational analysis document in 2009, Saving Newborn Lives in Nigeria: Newborn Health in the Context of the Integrated Maternal, Newborn and Child Health Strategy, as a feasible and low-cost	• There is a plan to develop and finalise KMC guidelines in 2017, according to the national milestones listed in the NiENAP. Currently the FMOH, in collaboration with the Maternal and Child Survival Program (MCSP), is	• The national operational KMC Guidelines were finalised and approved by the FMOH but have not been disseminated.

	approach to reduce mortality among preterm/LBW babies.[6] The analysis also called attention to the need for a national KMC policy and a routine data collection system.<br>• Another situational analysis conducted in 2011, the second edition of *Saving Newborn Lives in Nigeria: Newborn Health in the Context of the Integrated Maternal, Newborn and Child Health Strategy*, highlighted the progress of practicing KMC at various facility levels and reiterated the need for a national KMC policy, service guidelines, and routine data collection systems.[7]	developing national operational KMC Guidelines within the MCSP.	
**Country Support/Implementation**			
Levels and types of facilities implementing KMC	Lagos University Teaching Hospital started providing KMC services in 1990, although there might have been other teaching hospitals providing KMC services at the time. The US Agency for International Development's ACCESS program reintroduced KMC in the northern states of Kano and Zamfara in 2007.	• In an assessment led by Save the Children in 31 states, 202 facilities out of 757 (27%) reported availability of KMC services. However, differing definitions of what constitutes KMC might have caused the overestimation of this figure.[8]<br>• According to the assessment, most facilities that provide KMC are general hospitals (61%), followed by tertiary and specialist hospitals (16%). Some secondary health facilities carry out KMC. The primary-level health facilities do not provide KMC services, but they refer preterm babies to higher level facilities where babies are initiated in KMC.[8]<br>• Through MCSP, 14 KMC spaces have been established in 14 health facilities in Ebonyi and Kogi states.[9]	• The National Hospital in Abuja extended its neonatal healthcare unit to designate a space for KMC services.<br>• Some general, tertiary and specialist hospitals, as well as some secondary health facilities provide KMC, but only tertiary/teaching facilities seem to be scaling up KMC.<br>• The number of tertiary/teaching hospitals practicing KMC alongside incubator care has continued to increase as more members of the Nigerian Society of Neonatal Medicine (NISONM) are trained on KMC during the annual general and scientific meetings.
Percentage of LBW newborns		• Skin-to-skin care has not been adopted as a policy in Nigeria.	

initiated in facility-based KMC		• Less than10% of babies are placed in skin-to-skin contact with the mother right after birth according to an assessment done at Lagos Teaching Hospital. Babies are only placed in skin-to-skin contact when paediatricians are present and insist on it.<br>• According to a quality-of-care assessment conducted in Kogi and Ebonyi states, 13% of babies are placed in skin-to-skin contact right after birth.	
Funding	The FMOH *in Saving Newborn Lives in Nigeria: Situation Analysis and Action Plan for Newborn Health in the Context of the Integrated Maternal, Newborn and Child Health Strategy* in 2011 committed to create a budget line for the creation of six zonal KMC centers as part of the comprehensive newborn care strategy to achieve the Millennium Developmental Goals.[7] However, as of 2017, this had not been achieved.	Though the FMOH has been engaged in the scale-up of KMC, most funding for KMC continues to come from donors.	• KMC funding is a combination of donor and government monies.<br>• Funds for KMC have been allocated through the Programme for Results/Saving one Million Lives initiative funded by government through a World Bank loan disbursed to all the states.
**Research**			
Major or program-based studies currently being conducted related to KMC	The first Nigerian scientific study comparing KMC with conventional care was undertaken in three hospitals in Lagos in 2001.[10]	• Implementation of KMC by health workers in Nigeria identified the need to institute KMC policies at hospitals and to designate KMC wards to improve KMC implementation.[11]<br>• Save the Children led an assessment of facility-based care of small newborns in 2017.[8]	• Awareness and Perception of Kangaroo Mother Care Among Mothers and the Role of the Healthcare Providers in Selected Primary Healthcare Facilities in Calabar Local Government Area of Cross River State, Nigeria.[12]<br>• Knowledge and Attitude of Nigerian Health Workers Concerning Kangaroo Mother Care.[13]
**Knowledge Management**			
Centers of excellence or	KMC was introduced in Nigeria in the late 1990s in the Lagos University Teaching Hospital.	• There are three centers of excellence for KMC: Lagos	

state-of-the-art facilities for KMC/care of LBW babies		University Teaching Hospital, Obafemi Awolowo University Teaching Hospital (Ile-Ife), and Federal Medical Center (Katsina).	
KMC manuals, trainings and campaigns	• The FMOH, with the help of partners adapted a *KMC Training Manual* in 2008.[14]<br>• In 2010, there was a national KMC training for neonatologists/paediatricians in Lagos; the practicums sessions were conducted at the KMC ward of Lagos University Teaching Hospital.<br>• In 2011, there was a training of frontline health workers on KMC conducted by Save the Children and FMOH in Kaduna state.	• The Essential Newborn Care Course (ENCC) training package, which includes KMC, was launched in 2016. Also, through MCSP, 949 people have been trained in maternal newborn health practices, including KMC, since March 2014.[9] MCSP also established KMC centers in Kaduna State with Johnson & Johnson funds, and other partners supported the establishment of KMC centers.	
**Monitoring and Evaluation**			
KMC indicators included in the national HMIS	In 2013, two KMC indicators were included in the HMIS:<br>• Percentage of newborns initiated in facility-based KMC<br>Percentage of newborns initiated in facility-based KMC who were discharged according to criteria, left against medical advice, were referred out or died before discharge.	The FMOH is reviewing a proposal to include new indicators and to modify existing indicators in the HMIS. For KMC, the new indicators and modifications are as follows:<br>• Percentage of newborns identified as < 2,000 g (new indicator).<br>• Percentage of facilities with inpatient maternity services with operational KMC (new indicator).<br>• Include the number of live births < 2,000 g as a denominator to calculate the percentage of newborns initiated in facility-based KMC (modification).<br>The new indicators include the number of live births initiated in facility-based KMC as a numerator to calculate the percentage of newborns initiated in facility-based KMC. Currently, only the numerators of number of babies	The FMOH approved including a new KMC indicator and to modify an existing indicator in the HMIS:<br>• Percentage of newborns identified as < 2,000 g (new indicator).<br>• Include the number of live births < 2,000 g as a denominator to calculate the percentage of newborns initiated in facility-based KMC (modification).

		discharged from KMC, number left against medical advice, number referred out after initiation and those who died after initiation are recorded.	
KMC data recorded at health facilities	KMC was introduced in the facility post register in 2013. It collected information about the number of babies who were initiated in KMC. The recording of data was done manually.	• Data for the KMC indicators are collected and summarised by the health facilities. Summarised information is entered electronically into the DIHS2.<br>• Regarding KMC services, there is no tracking process to monitor whether KMC services are operational at the national level, but facilities that provide KMC services under specific programs use paper-based KMC registries. It was proposed that the FMOH should include an indicator in the HIMS about the percentage of facilities with inpatient maternity services with operational KMC. The FMOH is reviewing the proposal about the new and modified indicators.	
**Advocacy**			
Professional organizations that endorse KMC	• The Paediatric Association of Nigeria's (PAN) provided KMC training sessions at the PAN conference in 2010.<br>• NISONM has been incorporating KMC in its annual general and scientific meeting preconference workshops and community linkage programs.	• PAN and NISONM conduct trainings at the state level using the Essential Newborn Care package, which includes KMC.<br>• The Neonatal Subcommittee of the National Child Health Working Group, with members from PAN and NISONM, advocates for KMC. NISONM recently developed the *Newborn Discharge Guide: Help Your Baby Survive*, which includes KMC guidance.[15]	

		• KMC mentorship is provided through the Voluntary Paediatric Services Scheme.<br>• NISONM endorsed the global recommendations of KMC at its 10th Annual General and Scientific Meeting in July 2017.	
Champions	• Prof. Angela Okolo has been practicing KMC at the University of Benin Teaching Hospital using special KMC reclining chairs.<br>• Dr. Abimbola Williams, senior newborn advisor for Save the Children Nigeria, has been supporting KMC activities among paediatricians and neonatologists.<br>• Professor Chinyere Ezeaka was the co-director of the frontline health workers' KMC trainings conducted by Save the Children and the FMOH in Kaduna state in 2011.	• There are many local champions with a track record of promoting KMC.<br>• Professor Ezeaka is supporting the development of the KMC operational guidelines. She continues to advocate for KMC as the head of the neonatal unit at Lagos University Teaching Hospital and as the president of NISONM.<br>• Catherine Oluwatoyin Ojo, a chief nursing officer for the Special Baby Care Unit of Ahmadu Bello University Teaching Hospital, received the International Confederation of Midwifes/Save the Children EVERY ONE Midwife Award in 2011 for her work in maternal and newborn health, which includes teaching families about KMC.<br>• Professor Ebun Adejuyigbe established a standard KMC center in Ile-Ife.<br>• Dr. Suleiman Bello of Federal Medical Center, Keffi led the care of a small-baby study by Save the Children and established a KMC center in Katsina.	• Some local champions have successfully advocated within their institutions to designate a space for KMC services. Others, have worked at the national level to include KMC in NBH training guidelines and into other policy documents.

Table 2. Demographic and Health Survey (DHS) proxy indicators for kangaroo mother care

DHS Indicators Related to KMC		Nigeria DHS, 2013[16]	Nigeria DHS, 2018[17]
**Identification of LBW Babies**	**Characteristic**	**Percentage**	**Percentage**
Percentage distribution of live births in the 3 years preceding the survey by mother's estimate of baby's size at birth, according to background characteristics	*Very small*	4.4	2.8
	Smaller than average	10.5	10.9
Percentage of births that have a reported birthweight		N/A	16.4
Percentage of babies weighing less than 2.5 kg among births with a reported birthweight		N/A	8.1
**Initial Breastfeeding Indicators**		**Percentage**	**Percentage**
Percentage of children born in the 2 years preceding the survey who started breastfeeding within 1 hour of birth		33.2	42.1
Percentage of children born in the 2 years preceding the survey who started breastfeeding within 1 day of birth		73.7	82.3
**Skin-to-Skin Contact Indicator**		**Percentage**	**Percentage**
Percentage of births that had skin-to-skin contact for the most recent live birth in the 3 years preceding the survey		N/A	12.0

CHALLENGES

- One of the major challenges to accelerate the uptake of KMC in Nigeria is that many health providers are not aware of policies and guidelines that include KMC.
- There are funding needs for the training of community health workers, the designation of KMC spaces in health facilities, and dissemination of KMC information through media outlets.
- While hospitals collect information about KMC services, the collected data are not used. There is need to use KMC data for decision-making and for finding a more formal way to disseminate lessons learned.
- As in most countries, there is a need to strengthen the ambulatory follow-up of small babies.

LESSONS LEARNED

- Collaboration among the FMOH, partners and local champions for KMC has been important for integrating KMC into national policies.
- The formation of a neonatal subcommittee has been critical to the acceleration of KMC.

FUTURE ACTIONS

- Ensure harmonisation of KMC indicators and inclusion of the indicators in DHIS2.
- Advocate for free maternal and child health services to increase access nationally.
- Scale up the national health insurance program to include mothers in the states and communities.
- Establish the six zonal centers of excellence.
- Operationalise the Newborn Health Committee (a subcommittee of the Child Health Working Group of the FMOH) to support KMC scale-up.

DOCUMENTS AND RESOURCES

Document Title	Link to Document
Saving Newborn Lives in Nigeria: Newborn Health in the Context of the Integrated Maternal, Newborn and Child Health Strategy (2009)	http://www.healthynewbornnetwork.org/hnn-content/uploads/situation-action-plan-nigeria.pdf
Saving Newborn Lives in Nigeria: Newborn Health in the Context of the Integrated Maternal, Newborn and Child Health Strategy (2011)	http://countdown2030.org/documents/countdown-news/nigeria-full-report.pdf
Nigeria Every Newborn Action Plan: A Plan to End Preventable Newborn Deaths in Nigeria (2016)	https://www.healthynewbornnetwork.org/hnn-content/uploads/2033-Nigeria-Every-Child-Action-Plan-151216.pdf
Kangaroo Mother Care Training Manual (2008)	https://www.healthynewbornnetwork.org/resource/kangaroo-mother-care-training-manual/
Implementation of kangaroo mother care by health workers in Nigeria (2016)	https://doi.org/10.4314/njp.v43i4.4
An assessment of maternal, newborn and child health implementation studies in Nigeria: implications for evidence informed policymaking and practice (2016)	https://www.ncbi.nlm.nih.gov/pmc/articles/PMC5002878/
Reproductive, Maternal, Newborn, Child, Adolescent Health and Nutrition (RMNCAH+N) 2017-2019	https://www.globalfinancingfacility.org/sites/gff_new/files/documents/Nigeria-Investment-Case.pdf
Second National Strategic Health Development Plan 2018-2022	https://health.gov.ng/doc/NSHDP%20II%20Final.pdf

REFERENCES

1. Nigeria FMOH. *Reproductive, Maternal, Newborn, Child, Adolescent Health and Nutrition (RMNCAH+N)*. Abuja: Nigeria FMOH; c2017.
2. Nigeria FMOH. *Second National Strategic Health Development Plan 2018–2022*. Abuja: Nigeria FMOH; c2018.
3. Nigeria Federal Ministry of Health (FMOH). *Integrated Maternal Newborn and Child Health Strategy*. Abuja: Nigeria FMOH, c2007.
4. Nigeria FMOH. *Essential Newborn Care: A Guide for Essential Practice.* Abuja: Nigeria FMOH; c2014.
5. Nigeria FMOH. *Nigeria Every Newborn Action Plan: A Plan to End Preventable Newborn Deaths in Nigeria*. Abuja: Nigeria FMOH; c2016.
6. Nigeria FMOH, Save the Children, ACCESS. *Saving Newborn Lives in Nigeria: Newborn Health in the Context of the Integrated Maternal, Newborn and Child Health Strategy*. Abuja: Nigeria FMOH; c2009.
7. Nigeria FMOH, Save the Children, Jhpiego. *Saving Newborn Lives in Nigeria: Newborn Health in the Context of the Integrated Maternal, Newborn and Child Health Strategy*. 2nd ed. Abuja: Nigeria FMOH; c2011.
8. Save the Children, MCSP, Nigeria FMOH. *Assessment of Facility-Based Care of Small Newborns in Nigeria. Phase 1: Landscape Survey.* Washington, DC: Save the Children; c2017.
9. Maternal and Child Survival Program (MCSP). *Nigeria MNCH Country Summary, March 2017*. Baltimore, MD: Jhpiego; c2017.
10. Ibe OE, Austin T, Sullivan K, Fabanwo O, Disu E, Costello AM. A comparison of kangaroo mother care and conventional incubator care for thermal regulation of infants < 2000 g in Nigeria using continuous ambulatory temperature monitoring. *Ann Trop Paediatr*. 2004;24(3):245–251. doi:10.1179/027249304225019082.
11. Onubogu UC, Okoh BA. Implementation of kangaroo mother care by health workers in Nigeria. *Nigerian J Paediatr*. 2016;43(4):252–257. doi:10.4314/njp.v43i4.4.

12. Nsemo, AD, Mgbekem M. Awareness and perception of kangaroo mother care among mothers and the role of the healthcare providers in selected primary healthcare facilities in Calabar Local Government Area of Cross River State, Nigeria. *Global Journal for Research Analysis*. 2018;7:515–519.
13. Onubogu UC, Okoh BA. Knowledge and attitude of Nigerian health workers concerning kangaroo mother care. *International Journal of Tropical Disease and Health*. 2018;31(4):1–10. doi:10.9734/IJTDH/2018/42974
14. Nigeria FMOH. *Kangaroo Mother Care Training Manual*. Abuja: Nigeria FMOH; c2008.
15. Nigerian Society of Neonatal Medicine (NISONM). *Newborn Discharge Guide: Help Your Baby Survive*. Abuja: NISONM; c2016.
16. National Population Commission (NPC) [Nigeria] and ICF International. Nigeria Demographic and Health Survey 2013. Abuja: NPC and ICF International; c2014.
17. National Population Commission (NPC) [Nigeria] and ICF. Nigeria Demographic and Health Survey 2018. Abuja: NPC and ICF; c2019.

KANGAROO MOTHER CARE IN ETHIOPIA

OVERVIEW

Kangaroo Mother Care (KMC) was first introduced in Ethiopia in 1996 at the Black Lion Hospital. Since then, KMC services have been expanded to other hospitals and health facilities at all levels. Recently, KMC was included in a series of policy documents issued by the Federal Ministry of Health (FMOH): *The Newborn and Child Survival Strategy* 2015/16-1019/20[1], the *Health Sector Transformation Plan*[2], and the *Ethiopian National Healthcare Quality Strategy*.[3] The KMC target in these policies was set to reach 80% of preterm babies with KMC by the year 2020. Despite the emphasis that the government has put on reducing neonatal mortality by using evidence-based strategies, such as KMC, the number of preterm/low birth weight (LBW) newborns initiated in KMC remains low.

Table 1. Status of KMC in Ethiopia by strategic area

Domain	Prior to and during 2014	2015-2017	2017-2019
**Policy**			
National Health Policy	The Federal Ministry of Health (FMOH) drafted a policy and a plan to integrate kangaroo mother care (KMC) into the national coordination mechanism of newborn and child healthcare.[4]	• KMC was integrated into the *National Strategy for Newborn and Child Survival* in Ethiopia for 2015/16-2019/20[1] as part of the package of high-impact child survival interventions along the continuum of care.<br>• KMC is also covered in the *Health Sector Transformation Plan* (HSTP), which aims to improve equity, coverage and utilisation of health services, and to improve the quality of healthcare.[2] Building on the HSTP the *Ethiopian National Healthcare Quality Strategy* 2016-2020[3] was developed to improve the quality of prioritised interventions such as KMC.	• KMC will be included in the revisions of the HSTP and the *National Strategy for Newborn and Child Survival*.[1]<br>• The MOH and WHO released a call for proposals in 2017 to address some of the gaps in the existing policies that integrate KMC into newborn care, such as training, mentoring, and overall scale-up. The purpose of the proposal was to find efficient ways to scale up KMC in Ethiopia. The implementation research to scale up KMC has been ongoing through different stakeholders (universities and other partners). Data collection has been finalised. The drafting of the report is ongoing.
National Guidelines	• The first edition of the Standard Treatment Guidelines for district hospitals included an annex on KMC.[5]<br>• There are national guidelines about the care of		• There have not been any updates to the guidelines.

	preterm/LBW babies that include inpatient KMC. These guidelines were adopted from American Academy of Pediatrics (AAP) manuals for newborn care.		
**Country Support/Implementation**			
Levels and types of facilities implementing KMC	Black Lion Hospital started providing KMC services in 1996. KMC was later expanded to seven hospitals.	• All teaching hospitals and most general hospitals in Ethiopia are expected to provide KMC services. However, KMC is provided at some tertiary-, secondary- and primary-level facilities, and some private hospitals. Initiation of facility-based KMC remains low.<br>• Save the Children led an assessment between 2014 and 2015 about the delivery of KMC in six hospitals and 19 health centers across three zones (East Shewa in Oromia Region and Sidama and Gurage in SNNP Region). All six hospitals reported providing KMC services within the past 3 months of the assessment. However, only 14% of babies born at the surveyed hospitals who weighed less than 2,000 g were documented as enrolled into KMC, suggesting low levels of KMC initiation and identification of small newborns.[6]	• It is expected that the findings from the implementation research will inform the strategy to scale-up KMC. The MOH and WHO have facilitated workshops to discuss the preliminary findings among stakeholders.
Percentage of LBW newborns initiated in facility-based KMC		• About 10% to under 25% of preterm and/or LBW babies receive KMC in Ethiopia. This estimate is derived from an assessment that was conducted in two hospitals that were providing KMC services in Ethiopia. After training, these hospitals provided KMC services to 36% of preterm babies.	• A study that used 2016 data to assess the quality of KMC services in Ethiopia among 3,804 facilities found that out of 768 LBW babies, only 356 (46.6%) received KMC. Of those 66.7% survived and 12.9% died. The high mortality rate may indicate inadequate KMC supervision and follow-up.[7]

		• A KMC indicator has been included in the health management information system (HMIS). It is expected that there will be national-level data about the percentage of preterm babies who are initiated in KMC.	
Funding		Funding for KMC is mostly provided by the FMOH. There is a need for partners to support KMC.	Funding for KMC continues to be a combination of FMOH and partners' funding.
**Research**			
Major or program-based studies currently being conducted related to KMC		• WHO is leading a multicountry KMC implementation research study to determine effective models of delivery for KMC. The study is ongoing in four regions in Ethiopia and will eventually inform national scale-up of KMC.<br>• Save the Children led a rapid assessment about availability and delivery of care to premature/LBW babies in three zones in Ethiopia. The key findings were the large unmet need for KMC, the paucity of KMC data, there might be gaps in terms of timely and accurate identification of LBW babies, and the top barriers to KMC provision were low volumes of LBW babies, lack of equipment and untrained staff.[8]<br>• Mothers' practice and associated factors on kangaroo mother care in Mekelle City Hospitals Tigray, North Ethiopia: a cross-sectional survey.[9]	• A community-based cross-sectional study conducted in 2017 in Yirgalem town among 215 mothers of preterm/LBW newborns found that 90 (41.9%) subjects practiced KMC, out of which 31 (14.4%) initiated KMC immediately after birth, and 59 (27.4%) after 24 hours. Giving birth spontaneously and delivering at a governmental hospital were factors associated with practicing KMC. The authors concluded that there is a need to promote the continuous practice of KMC at home while conducting studies about the acceptability and applicability of KMC to identify barriers to using KMC in Ethiopian homes.[10]<br>• Mersha et al. aimed to assess factors associated with neonatal sepsis in Wolaita Sodo Town. Their findings included that 229 (83.3%) newborns had been put in KMC position within 1 hour of being born. These newborns were 94% less likely to acquire sepsis than those who were not placed in KMC position.[11]

			• Quality of kangaroo mother care services in Ethiopia: implications for policy and practice.
**Knowledge Management**			
Centers of excellence or state-of-the-art facilities for KMC/care of LBW babies	Black Lion Hospital has been advocating for KMC for the past 20 years.	Currently, no facilities have been nominated as centers of excellence for KMC, but Black Lion Hospital continues to lead KMC advocacy efforts.	No information was provided on new facilities being nominated as centers of excellence for KMC.
KMC manuals, trainings and campaigns	• In 2009, the KMC *Trainee's Manual* was published.[12]<br>• The *Newborn Care Training Participants Manual*[12] recommended that all LBW babies with a weight below 2,000 g be referred to the nearest health facility with KMC services or to a higher level of care.	In 2015/16, a training manual for care of preterm/LBW babies which includes KMC was developed.	
**Monitoring and Evaluation**			
KMC indicators included in the national HMIS		In 2017, a KMC indicator was included in the HMIS to calculate the proportion of preterm/LBW babies for whom KMC was initiated.	KMC coverage figures are not available.
KMC data recorded at health facilities	Health facilities use the Integrated Supportive Supervision (ISS) checklist to report if they provide KMC services.	• Although admission to KMC services is included in the HMIS delivery register, KMC is not included on the reporting form, and registers are often incomplete.[8]<br>• There is a quality self-assessment for the facilities, which includes a KMC indicator.	

Advocacy			
Professional organizations that endorse KMC	The Ethiopian Paediatric Society, which is the lead professional association on newborn health matters in Ethiopia, has endorsed KMC as a high-impact newborn health intervention.		• The Ethiopian Paediatric Society has been engaged in establishing KMC spaces, equipping them, and training health workers.
Awareness campaigns		• In 2016, the MOH, regional health bureaus, and its partners raised awareness about KMC during World Prematurity Day under the motto "Kangaroo mother care is an effective method of treating premature babies."<br>• Annually during the month of November, newborn care, including KMC, is promoted through mass media.	
Champions	Professor Bogale Worku introduced KMC at the Black Lion Hospital.	Professor Worku, considered a global expert in KMC, has contributed to the evidence-based findings of KMC in Ethiopia.	

Table II. DHS Proxy Indicators for KMC

DHS Indicators Related to KMC (Ethiopia DHS, 2016)[14]		
**Identification of LBW babies**	**Characteristic**	**Percent**
Percentage distribution of live births in the 5 years preceding the survey by mother's estimate of baby's size at birth, according to background characteristics.	*Very small*	16
	Smaller than average	10
Percentage of births that have a reported birth weight		13.6
Percentage of babies weighing less than 2.5 kg among births with a reported birth weight		13.2
**Initial Breastfeeding**		**Percent**
Percentage of children born in the 2 years preceding the survey who started breastfeeding within 1 hour of birth		73.3
Percentage of children born in the 2 years preceding the survey who started breastfeeding within 1 day of birth		91.9
**Skin-to-Skin Contact**		**Percent**
Percentage of births that had skin-to-skin contact for the most recent live birth in the 3 years preceding the survey		24.3

CHALLENGES

- There are some gaps in training, mentoring, support provision and overall scale-up of KMC. Recently, the FMOH and WHO released a call for proposals to identify efficient ways to scale up KMC in Ethiopia.
- KMC activities lack funding partly due to the belief that there are no costs involved in KMC. However, resources are needed to conduct trainings, purchase supplies, designate a physical space for KMC in health facilities, assign a nurse to the KMC spaces, establish ambulatory follow-up services, etc.
- There is a gap in terms of education and mentoring of healthcare providers, given that some providers perceive KMC as an inferior alternative to incubators.
- There is a need to document the successes of KMC in Ethiopia.

LESSONS LEARNED

- Inclusion of KMC as a national target in the *Health Sector Transformation Plan* and in the *National Strategy for Newborn and Child Survival* has been critical in improving the availability and quality of KMC services in Ethiopia.
- Standardising the referral and follow-up of premature babies is important.

FUTURE ACTIONS

- Include KMC in the integrated systems strengthening supportive supervision checklist.
- Adapt the job aids from the WHO KMC implementation research sites to use for KMC scale-up.
- Ensure KMC is part of continuing medical education/continuing professional development for maternal and child health professional associations (Ethiopian Paediatrics Society, Ethiopian Society of Obstetricians and Gynaecologists, Ethiopian Midwives Association).

- Make sure KMC is adequately included during the revision process of maternal and child health training materials, such as those on basic and comprehensive emergency obstetric and newborn care, integrated management of newborn and childhood illnesses, and integrated community case management.
- Synthesise and use KMC data from the newly added health management information system data.

DOCUMENTS AND RESOURCES

Document Title	Link to Document
Standard Treatment Guidelines for District Hospitals, 1st ed. (2004).	http://collections.infocollections.org/whocountry/en/d/Js6852e/12.4.html
Newborn and Child Survival Strategy Document Brief Summary 2015/16-2019/20 (2015)	https://www.unicef.org/ethiopia/Child_Survival_Strategy.pdf
Health Sector Transformation Plan (2015).	https://www.researchgate.net/file.PostFileLoader.html?id=56efcc94cbd5c2c1930fae21&assetKey=AS%3A342023027609601%401458556052424
Ethiopian National Health Care Quality Strategy 2016-2020 (2015).	https://www.medbox.org/et-policies-others/ethiopian-national-health-care-quality-strategy-2016-2020/preview
Newborn Care Training Participants Manual (2012)	http://www.epseth.org/a/files/NBT%20manual%20Bogale%20august%202012.pdf
Kangaroo mother care: a randomized controlled trial on effectiveness of early kangaroo mother care for the low birth weight infants in Addis Ababa, Ethiopia (2005)	https://www.ncbi.nlm.nih.gov/pubmed/15840760
Rapid Health Facility Assessment on Service Availability and Delivery of Care to Premature and/or Low Birth Weight Babies (2016)	http://www.healthynewbornnetwork.org/hnn-content/uploads/KMC_Facilities_Brief_.pdf
Utilization of Utilization of Kangaroo Mother Care (KMC) and Influencing Factors Among Mothers and Care Takers of Preterm/Low Birth Weight Babies in Yirgalem Town, Southern, Ethiopia	http://diversityhealthcare.imedpub.com/utilization-of-utilization-of-kangaroo-mother-care-kmc-and-influencing-factors-among-mothers-and-care-takers-of-pretermlow-birth-w.pdf
Mothers' practice and associated factors on kangaroo mother care in Mekelle City hospitals Tigray, North Ethiopia: a cross-sectional survey	http://medicaljournals.stmjournals.in/index.php/RRJoI/article/view/1270
Neonatal sepsis and associated factors among newborns in hospitals of Wolaita Sodo Town, Southern Ethiopia	https://search.proquest.com/openview/4d101dcdd7fe493b767f91284f7d06a0/1?pq-origsite=gscholar&cbl=3933284

Quality of kangaroo mother care services in Ethiopia: implications for policy and practice (2019)	https://journals.plos.org/plosone/article?id=10.1371/journal.pone.0225258

REFERENCES

1. Ethiopia FMOH. *Newborn and Child Survival Strategy Document Brief Summary 2015/16–2019/20*. Addis Ababa: FMOH; c2015.
2. Ethiopia FMOH. *Health Sector Transformation Plan*. Addis Ababa: FMOH; c2015.
3. Ethiopia FMOH. *Ethiopian National Health Care Quality Strategy* 2016–2020. Addis Ababa: FMOH; c2015.
4. Ethiopia FMOH. *Making Kangaroo Mother Care a Universal Practice in the Country.* Addis Ababa: FMOH; c2014.
5. Drug Administration and Control Authority of Ethiopia (DACA). *Standard Treatment Guidelines for District Hospitals*. 1st ed. Addis Ababa: DACA; c2004.
6. Mathewos B, Sitrin D, Valsankar B, Musena Y, Tadesse L, Chan G, Tadesse Y. *Delivery of Kangaroo Mother Care in Ethiopian Facilities: Results from a Rapid Assessment in Three Zones*. Available from: https://www.healthynewbornnetwork.org/hnn-content/uploads/KMC_Facilities_Brief_.pdf. c2015.
7. Weldearegay HG, Medhanyie AA, Abrha MW, Tadesse L, Tekle E, Yakob B, et al. Quality of kangaroo mother care services in Ethiopia: implications for policy and practice. *PLoS ONE*. 2019;14(11):e0225258. doi:10.1371/journal.pone.0225258
8. Save the Children. *Rapid Health Facility Assessment on Service Availability and Delivery of Care to Premature and/or Low Birth Weight Babies*. Addis Ababa: Save the Children; c2016.
9. Solomon E, Fiseha G, Hailemariam B, et al. Mothers' practice and associated factors on kangaroo mother care in Mekelle City Hospitals Tigray, North Ethiopia: a cross-sectional survey. *Research and Reviews: Journal of Immunology*. 2016;6(1):21–31.
10. Yusuf E, Fiseha F, Dulla D, Kassahun G. Utilization of kangaroo mother care (KMC) and influencing factors among mothers and care takers of preterm/low birth weight babies in Yirgalem Town, Southern, Ethiopia. *Diversity & Equality in Health and Care*. 2018;15. doi:10.21767/2049–5471.1000160.
11. Mersha A, Worku T, Shibiru S, Bante A, Addis M. Neonatal sepsis and associated factors among newborns in hospitals of Wolaita Sodo Town, Southern Ethiopia. *Res Reports Neatol*. 2019;9:1–8. doi:10.2147/RRN.S193074.
12. John Snow Inc., Ethiopia FMOH, Save the Children. *Kangaroo Mother Care Trainee's Manual*. Boston: John Snow; c2009.
13. Ethiopia Federal Ministry of Health (FMOH). *Newborn Care Training Participants Manual*. Addis Ababa: FMOH; c2012.
14. Ethiopia Central Statistical Agency (CSA) and ICF. *Ethiopia Demographic and Health Survey* 2016. Rockville, MD: ICF; c2016.

KANGAROO MOTHER CARE IN INDIA

OVERVIEW

The Government of India is committed to improving child health by prioritising newborn care services that increase child survival. In 2014, the Child Health Division of the Ministry of Health and Family Welfare (MOHFW) released the Kangaroo Mother Care and Optimal Feeding of Low Birth Weight Infants: Operational Guidelines in an effort to implement kangaroo mother care (KMC) at the facility level.[1] Another document, published in 2014, that included KMC was the India Newborn Action Plan (INAP)[2], which highlighted KMC as a specific intervention recommended for small and sick newborns who weigh less than 2,000 g. One of the priority actions outlined in the INAP for KMC was the establishment of fully functional KMC units/wards in health facilities that provide newborn care services. The MOHFW allotted funds to each state for the adaptation of KMC spaces within the special newborn care units. From 2016 to 2019, the number of KMC units almost tripled from 265 to 782.

As more health facilities are expected to provide KMC services, data collection will be critical to monitor quality, identify gaps, and track services. Engagement of healthcare providers and the identification of champions will help to foster the ownership of KMC as an effective intervention, speeding up the rate at which KMC services are adopted throughout the country.

Table I. Status of KMC in India by strategic area

Domain	Prior to and during 2014	2015-2017	2017-2019
**Policy**			
National Health Policy	In 2014, kangaroo mother care (KMC) was included in the India Newborn Action Plan (INAP) as a specific intervention recommended for reducing morbidity and mortality among small and sick newborns. One of the priority actions outlined in the INAP was the establishment of fully functional sick newborn care units (SNCUs) with attached KMC units/wards. The KMC coverage targets were set at 35% by 2017, 50% by 2020, 75% by 2025 and 90% by 2030.[2]	• The 2017 National Health Policy of India gives highest priority to reducing newborn deaths in the country.<br>• KMC was initiated as a pilot in a few sites in the country. Preliminary results demonstrated scalability and acceptance. Additionally, the MOHFW piloted an integrated approach to newborn care at the facility level through the Family-Participatory Care (FPC) model, which was taken for scale-up at all district SNCUs across India. The FPC model focuses on families as participants in newborn care, which includes skin-to-skin contact, breastfeeding, and identification of	• KMC has been included in the operational guidelines for Family Participatory Care for Improving Newborn Health.[3]

		danger signs, all of which are components of KMC.	
National Guidelines	The Kangaroo Mother Care and Optimal Feeding of Low Birth Weight Infants: Operational Guidelines include eligibility criteria for KMC, specifications for infant feeding, infrastructural requirements to establish a KMC ward, an institutionalisation plan, monitoring plan, budgetary guidelines and a communication strategy. Additionally, facility and community guidelines for KMC are included in several other training packages being used to train health personnel on newborn care.[1]		• The MOHFW and the Ministry of Women and Child Development launched the Home-Based Care for Young Child (HBYC) programme with the goal of reducing child mortality and morbidity by improving nutrition, growth, and early child development. The HBYC includes KMC in the LBW and sick infants section.[4]
**Country Support/Implementation**			
Levels and types of facilities implementing KMC	• The Government of India (GOI) aims to establish national, regional and state newborn resource centers as centers of excellence for newborn care practices, including KMC. These centers will provide technical support to SNCUs and newborn stabilisation units to initiate KMC services.	• According to the minutes of the KMC Acceleration Partnership 2016 meeting, there were 630 SNCUs in India. The number of SNCUs has since increased to 712. Of these, 265 reported having a KMC unit. However, only 15% have the recommended number of eight beds per unit.<br>• In April 2017, GOI created a technical advisory group to recommend and support a strategy for scaling up KMC across the country. Two working groups have been constituted, led by the KMC Foundation, Gujarat, and the Postgraduate Institute of Medical Education and Research in Chandigarh, to develop KMC	• As of March 2019, there were 848 functional SNCUs of which 782 at district hospitals reported having provided KMC services.<br>• In India, KMC is recommended for small and sick babies. However, medical colleges and tertiary hospitals are the only health facilities initiating sick babies in KMC.<br>• All tertiary hospitals/medical colleges also provide KMC services.<br>• GOI recommended that states develop mother newborn care units (MNCUs) which will serve to decongest the SNCUs. The MNCUs should have an area designated for KMC.

		training modules and reporting tools. This work is in progress.	• Currently, all states and UTs report on the status of KMC activities online.
Percentage of LBW newborns initiated in facility-based KMC		There is no mechanism to collect data on KMC parameters at present. Data were submitted to the GOI by 18 states, which revealed that KMC was provided to 0-20% of SNCU-admitted babies in 12 states and more than 20% of SNCU-admitted babies in six states.	• In 2019, it was estimated that 10-<25% of preterm and/or LBW babies are initiated in facility-based KMC, as per the SNCU online system.
Funding		• The KMC guidelines of the MOHFW contain a section on infrastructural and human resources requirements to establish KMC wards. The suggested budget is INR 266,100 (US$ 4,119) for essential items.[1] Budgetary provisions have been made by the GOI in the annual health plans submitted by states to the GOI, and states have been informed of the federal management regulation head under which various components for establishing KMC wards can be budgeted.<br>• Donor funding for KMC continues, with US Agency for International Development funds being channelled through Save the Children, John Snow Inc., IPE Global and others. Norwegian funds are being channelled through the Norway India Partnership Initiative for establishing models of FPCs. UNICEF funding is utilised to coordinate efforts for developing KMC training materials and tools.	• Funding for KMC is a combination of donor and MOH funding.<br>• MOH provides funds for the implementation of KMC guidelines at all the SNCUs. However, there is a need to fund research projects and technical support.

		• A budget of INR 100,000 (US$ 1,548) was sent to each district to ensure that KMC units were made an integral part of the SNCUs.[5]	
**Research**			
Major or program-based studies currently being conducted related to KMC	• Experience with kangaroo mother care in a neonatal intensive care unit (NICU) in Chandigarh, India.[6]	The World Health Organization is conducting a multicentric study on the feasibility of KMC for unstable babies. Two other studies are being conducted with research grants in the state of Haryana by the Society for Applied Studies and the Community Empowerment Lab in Uttar Pradesh. Studies have been undertaken by India's premier research body, the Indian Council of Medical Research, on community KMC. Several smaller research studies have been undertaken by the KMC Foundation of India and other medical colleges located in the states of Gujarat, Maharashtra, Tamil Nadu and Andhra Pradesh.<br><br>Some papers published on KMC practices include:<br>• "The presence of physician champions improved kangaroo mother care in rural western India."[7]<br>• "Rolling out of kangaroo mother care in secondary level facilities in Bihar–some experiences."[8]<br>• "Efficacy of skilled based teaching program on kangaroo mother care among postnatal mothers in a rural	• The study "Effect of community-initiated kangaroo mother care on survival of infants with low birthweight: a randomized controlled trial" published in the Lancet in 2019 concluded that community-initiated kangaroo mother care substantially improves newborn baby and infant survival. Some 8,402 babies were enrolled between 2015 and 2018; 4,480 were assigned to the intervention group and 3,922 to the control group. Between enrolment and 28 days, 73 infants died in the intervention group and 90 in the control group. Between enrolment and 180 days (hazard ratio [HR] 0.70, 95% CI 0.51–0.96; $p = 0.027$), 158 infants died in the intervention group and 184 infants in the control group (HR 0.75, CI 0.60–0.93; $p = 0.010$).[10]

		tertiary care teaching hospital of central India."[9]	
**Knowledge Management**			
Centers of excellence or state-of-the-art facilities for KMC/care of LBW babies	KMC was introduced in 1994/95 in BJ Medical College and Hospital in Ahmedabad, Gujarat. King Edward Medical College (KEMC) in Mumbai and the All India Institute of Medical Sciences (AIIMS) in New Delhi soon followed.<br>Centers of excellence were established from 2003-2005 at the Postgraduate Institute of Medical Education and Research in Chandigarh, KEMC, Institute of Child Health and Hospital for Children in Chennai, Kalawati Saran Children's Hospital in New Delhi, AIIMS, and BJ Medical College.	The five centers of excellence that were established from 2003-2005 continue to be centers of excellence today. Additionally, there is a plan in progress to set up model KMC services in 25 regional and state resource centers by the end of 2017.	No other KMC centers of excellence have been established.
KMC manuals, trainings and campaigns	In 2006-2011, there were KMC training workshops at centers of excellence during newborn week. In 2009, a training workshop on KMC was held at the annual conference of the National Neonatology Forum (NNF). The IX International Conference on KMC was hosted in India in Hyderabad, Andhra Pradesh.	• A working group was established at the request of the MOHFW. This group is developing KMC training manuals for health providers.<br>• AIIMS developed smartphone apps for continuing education for the management of care for sick newborns based on standard treatment protocols, which include KMC components.<br>• International KMC conferences were organised in India in 2009, 2012 and 2016.	• KMC training modules are still being developed.<br>• The Handbook for Accredited Social Health Activists (ASHAs) on Home Based Care for Young Child (HBYC), lists KMC as a strategy that ASHAs should recommend to families with LBW and/or sick infants. In addition to the six home visits within 42 days after discharge of LBW or sick babies that ASHAs perform, five additional visits have been added after the 42nd day. The handbook specifies that KMC helps to provide warmth through skin-to-skin contact, to provide closeness to the mother's breast and to prevent hypothermia. It is also highlighted that fathers can

			also practice the skin-to-skin component of KMC.[4]
**Monitoring and Evaluation**			
KMC indicators included in the national HMIS		• Currently, there are no KMC indicators in the HMIS. The SNCU online software is the portal for data entry on SNCU activities, and it contains a yes/no indicator on KMC. However, it is not yet very reliable. As mentioned above, the GOI has constituted working groups to develop more appropriate indicators and mechanisms for data recording and reporting. Once finalised, these will be included in HMISs throughout the country.	Six KMC indicators were developed and integrated into the SNCU online system. To date, 782 SNCUs have reported on KMC indicators monthly.<br>• Number of newborn admitted to SNCU receiving KMC.<br>• Average duration of KMC.<br>• Percentage of health facilities with SNCUs that have operational KMC units.<br>• Percentage of newborns below 2,000 g who continued KMC until discharge.<br>• Percentage of preterm infants (gestation age less than 37 weeks) delivered in the health facility.<br>• Percentage of preterm newborns who received KMC until 40 weeks (expected delivery date or full term).
**Advocacy**			
Professional organizations that endorse KMC		• The NNF, Indian Academy of Paediatrics, Indian Association of Neonatal Nurses, Federation of Obstetric and Gynaecological Societies of India, and Trained Nurses' Association of India are some of the professional organizations comprising paediatricians, gynaecologists and nurses that endorse KMC. These organizations promote KMC through sessions in conferences and workshops. The NNF developed	

		accreditation guidelines for newborn units in the public and private sector, which include KMC services. A guideline and communication tool on KMC were developed by the NNF and are featured on its website.<br>• The KMC Foundation was formed after the IX International Conference of KMC in India to scale up awareness, advocacy and adequate practice of KMC.	
Awareness campaigns		KMC is part of training programs. Although communication materials have been developed and are available for download from the GOI website, KMC has not yet been advertised or promoted through multimedia on a large scale.	• GOI has developed and disseminated SBCC materials including posters, job aids and training videos to promote KMC.
Champions	• Dr. Ashok Deorari, trainer<br>• Dr. Vinod Paul, head of paediatrics department for AIIMS, pushing newborn care interventions	• There are local champions with limited resources. Most of them are clinicians and practitioners, including Professor Shashi N. Vani, Dr. Rekha Udani, Professor Sushma Nangia, Professor Suman Rao, Dr. Ashok Deorari of AIIMs, and Dr. Ruchi Nanavati of King Edward Memorial Hospital and Seth GS Medical College in Mumbai.	• Champions have been strongly advocating through conferences and publications and by conducting nationwide KMC trainings.

Table 2. National Family Survey (NFHS) proxy indicators for kangaroo mother care

DHS Indicators Related to KMC		(NFHS-4) 2015-16[11]
**Identification of LBW Babies**	**Characteristic**	**Percentage**
Percentage distribution of all live births in the 5 years preceding the survey by mother's estimate of baby's size at birth, according to background characteristics	*Very small*	3.1
	Smaller than average	9.0
Percentage of live births in the 5 years preceding the survey with a reported birthweight		77.9
Percentage of babies weighing less than 2.5 kg among births with a reported birthweight		18.2
**Initial Breastfeeding Indicators**		**Percentage**
Percentage of children born in the 2 years preceding the survey who started breastfeeding within 1 hour of birth		41.5
Percentage of children born in the 2 years preceding the survey who started breastfeeding within 1 day of birth		81.4
**Skin-to-Skin Contact Indicator**		**Percentage**
Percentage of births that had skin-to-skin contact for the most recent live birth in the 3 years preceding the survey		N/A

CHALLENGES

- One of the major challenges in accelerating KMC uptake has been the motivation of healthcare providers to practice and advocate for this initiative.
- Increasing KMC coverage depends on designating spaces for KMC in the district hospitals in the sick newborn care units (SNCUs). There are funding gaps to cover the adaptation of KMC spaces in existing facilities and the costs of KMC spaces in new facilities.

LESSONS LEARNED

- The active role of the MOHFW has been critical in India: It designated two working groups to help to accelerate the scale-up of KMC services. One of these working groups is drafting KMC indicators that will be included in the health management information system.
- Effective data use for KMC is important for engaging health professionals in KMC programs.

FUTURE ACTIONS

- The GOI established a technical advisory group comprising two working groups that will develop capacity-building, monitoring and evaluation tools for KMC.
- Funding has been allocated for KMC, but more SNCUs will designate space for KMC and provide KMC services.
- The regional and state newborn resource centers will guide states in implementing KMC and maintaining its quality.

DOCUMENTS AND RESOURCES

Document Title	Link to Document
Kangaroo Mother Care and Optimal Feeding of Low Birth Weight Infants: Operational Guidelines (2014)	www.nrhmorissa.gov.in/writereaddata/Upload/Documents/Operational_GuidelinesKMC_&_Optimal_feeding_of_Low_Birth_Weight_Infants.pdf
India Newborn Action Plan (2014)	www.newbornwhocc.org/INAP_Final.pdf
KMC poster: Kangaroo Mother Care India Network	www.kmcindia.org/images/kmc-poster.pdf
Experience with kangaroo mother care in a neonatal intensive care unit (NICU) in Chandigarh, India (2009)	https://doi.org/10.1007/s12098-009-0024-2
The presence of physician champions improved kangaroo mother care in rural western India (2016)	https://doi.org/10.1111/apa.13445
Rolling out of kangaroo mother care in secondary level facilities in Bihar–some experiences (2016)	http://www.ijph.in/article.asp?issn=0019-557X;year=2016;volume=60;issue=4;spage=302;epage=308;aulast=Neogi
Efficacy of skilled based teaching program on kangaroo mother care among postnatal mothers in a rural tertiary care teaching hospital of central India (2016)	http://ajner.com/HTMLPaper.aspx?Journal=Asian%20Journal%20of%20Nursing%20Education%20and%20Research;PID=2016-6-3-9
Initiating kangaroo mother care in facilities in limited resource settings (2017)	https://www.sciencedirect.com/science/article/pii/S2214999617303223
Community based kangaroo mother care for low birth weight babies: a pilot study (2017)	https://doi.org/10.4103/ijmr.IJMR_603_15
Effect of community-initiated kangaroo mother care on survival of infants with low birthweight: a randomised controlled trial (2019)	https://www.thelancet.com/journals/lancet/article/PIIS0140-6736(19)32223-8/fulltext
Handbook for Accredited Social Health Activist on Home Based Care for Young Child (2019)	http://nhsrcindia.org/sites/default/files/Handbook%20for%20ASHA%20on%20Home%20Based%20Care%20for%20Young%20Child-English.pdf
Facility Based Newborn Care (FBNC) Programme with special focus on Special Newborn Care Units (SNCUs) 2007-2017	https://www.unicef.org/evaldatabase/files/SNCU_Evaluation_Final_Report_India-2019.pdf

REFERENCES

1.India MOHFW. *Kangaroo Mother Care and Optimal Feeding of Low Birth Weight Infants: Operational Guidelines*. New Delhi: MOHFW; c2014.

2.India Ministry of Health and Family Welfare (MOHFW). *India Newborn Action Plan*. New Delhi: MOHFW; c2014.

3.India MOHFW. *Family Participatory Care for Improving Newborn Health Operational Guidelines for Planning and Implementation*. New Delhi: MOHFW; c2017.

4.India MOHFW. *Handbook for Accredited Social Health Activist on Home Based Care for Young Child*. New Delhi: MOHFW; c2019.

5.Srivastava R. UP hospitals to fight infant mortality with mom's magic touch. *Hindustan Times*. September 7, 2016.

6.Parmar VR, Kumar A, Kaur R, et al. Experience with kangaroo mother care in a neonatal intensive care unit (NICU) in Chandigarh, India. *Indian J Pediatr*. 2009;76(1):25–28. doi:10.1007/s12098-009-0024-2.

7.Soni A, Amin A, Patel DV, et al. The presence of physician champions improved kangaroo mother care in rural western India. *Acta Paediatr*. 2016;105(9):e390–e395. doi:10.1111/apa.13445.

8.Neogi SB, Chauhan M, Sharma J, Negandhi P, Sethy G. Rolling out of kangaroo mother care in secondary level facilities in Bihar–some experiences. *Indian J Public Health*. 2016;60(4):30–38. doi:10.4103/0019-557X.195864.

9.Wasnik AM. Efficacy of skilled based teaching program on kangaroo mother care among postnatal mothers in a rural tertiary care teaching hospital of central India. *Asian Journal of Nursing Education and Research*. 2016;6(3):331–336. doi:10.5958/2349-2996.2016.00062.8.

10.Mazumder, Sarmila et al. Effect of community-initiated kangaroo mother care on survival of infants with low birthweight: a randomised controlled trial. *Lancet*. 2019;394(10210):1724–1736.

11.International Institute for Population Sciences (IIPS) and ICF. *National Family Health Survey (NFHS–4), 2015–16: India*. Mumbai: IIPS; c2017.

KANGAROO MOTHER CARE IN MALAWI

OVERVIEW

Malawi's implementation of kangaroo mother care (KMC) has progressed since its introduction as a pilot in 1999. The Ministry of Health (MOH), with the support of partner organizations, instituted policies and strategies that prioritized the care of small and preterm newborns. National guidelines for KMC were developed in 2005,[1] and in 2015 the MOH launched the country's Every Newborn Action Plan (ENAP), which set an ambitious goal of reaching 75% of eligible newborns with KMC by 2020 and 90% by 2035.[2] Facility-based KMC services are tracked through the national health information management system, which in 2015 was strengthened to include core indicators and standardised national registers, as well as reporting forms for KMC. Today, KMC continues to be highlighted in guidelines, trainings, and campaigns to reduce the number of preventable deaths among newborns. The World Health Organization and UNICEF featured Malawi's progress in reducing neonatal mortality rates in the report *Reaching Every Newborn National 2020 Milestones* (2017).[3] It is crucial that Malawi continues to move forward by increasing the coverage of KMC services, mentoring staff, improving KMC data quality and data use, and disseminating the lessons learned from the KMC centers of excellence.

Table 1. Status of KMC in Malawi by strategic area

Domain	Prior to and during 2014	2015-2017	2017-2019
**Policy**			
National Health Policy	Kangaroo mother care (KMC) was integrated into national policy in 2005 through one of several essential healthcare packages implemented by the Malawi Ministry of Health (MOH), as detailed in the *Road Map for Accelerating the Reduction of Maternal and Neonatal Mortality and Morbidity in Malawi.*[4]	In 2015, the MOH launched the Malawi Every Newborn Action Plan (ENAP), which emphasises strengthening the health systems to achieve the high coverage of interventions such as KMC. The coverage targets for facility-based KMC listed in the ENAP are 75% by 2020, 80% by 2025, 85% by 2030 and 90% by 2035.[2]	• KMC was included in the National Health Policy that was enacted in 2018.
National Guidelines	• The first national guidelines for KMC were published in February 2005 and revised in March 2009 to cover ambulatory and community KMC. The guidelines recommended that all babies less than 2,500 g be started on KMC and specified admission and discharge criteria for each facility level. In general, stable babies weighing 2,000-2,500 g are	KMC was incorporated into the Integrated Maternal and Newborn Care training package.	• The National Sexual and Reproductive Health and Rights (SRHR) Policy which includes KMC was updated in 2017.[5] It is planned that in 2022, this policy will be updated again.

	initiated in KMC at the health facility and then sent home. Mothers are given instructions on when to bring their baby back for follow-up.[1]<br>• KMC was included in the Child Health Strategy 2014-2020 as an intervention to avert neonatal deaths.[5]<br>• KMC was incorporated in the Malawi National Reproductive Health Service Delivery Guidelines 2014-2019. The guidelines specify that all babies with a birth weight of less than 2,500 g should be initiated in KMC.[7]		
**Country Support/Implementation**			
Levels and types of facilities implementing KMC	KMC was established in all four central hospitals and in the majority of district hospitals. In 2014, all 87 hospitals were assessed; 79% of hospitals reported providing inpatient KMC services.[8]	The majority of public hospitals implement KMC. The establishment of sick newborn care units in all the district hospitals is ongoing. However, there is a gap in coverage of KMC in private for-profit hospitals.	All government-owned tertiary and district hospitals offer facility-based KMC services, but it is difficult to estimate whether KMC is fully operational due to inconsistent reporting through the DHIS2.
Percentage of LBW newborns initiated in facility-based KMC	Using data from the 2014 emergency obstetric and newborn care (EmONC) survey, an analysis of KMC readiness showed that KMC initiation rates for all live births for facility deliveries at hospitals ranged from 0.6% to 17.4%.[8]<br>In 11 districts, it was estimated that in 2014 16% of preterm/LBW newborns were initiated in facility-based KMC.[9]	About 21% in 2015, 19% in 2016, and 18% in 2017 of preterm/LBW newborns were initiated in facility-based KMC in 11 districts in 2016.[9]	It is estimated that less than 10% of preterm/LBW newborns are initiated in facility-based KMC, according to data extracted from maternity reports within DHIS2. There are issues with data quality including inconsistencies and lack of reporting that might result in underestimation of the initiation rates.
Funding		Funding for KMC is a combination of donor and MOH funds. Government funds come as reproductive, maternal, newborn and adolescent health, and	Funding for KMC continues to be a combination of donor and MOH funds. Donor funds support training and mentorship/supervision activities, while

		they are not specific to intervention areas. However, most of the KMC capacity-building efforts and supplies are procured and distributed through partners, while government funding covers staff salaries. Funding gaps for KMC are mostly in creating neonatal units of care, increasing the quality of care of LBW newborns, and advocating to prioritize KMC.	staff working in KMC units and referral hospitals are paid with government funds.
**Research**			
Major or program-based studies currently being conducted related to KMC	Several studies and program-based learnings were conducted prior to or during 2014 including:<br>• Readiness of hospitals to provide kangaroo mother care (KMC) and documentation of KMC service delivery: analysis of Malawi 2014 emergency obstetric and newborn care (EmONC) survey data.[8]<br>• Evaluation of Kangaroo Mother Care Services in Malawi.[10]	There are a series studies being conducted on KMC. These include:<br>• Born too small: who survives in the public hospitals in Lilongwe, Malawi?[11]<br>• Investigating preterm care at the facility level: stakeholder qualitative study in central and southern Malawi.[12]<br>• Assessment of early outcomes among newborns discharged from facility-based KMC in three hospitals (SNL).<br>• Evaluation of the use of a customised wrap to improve the uptake of skin-to-skin practices (SNL/Save the Children Norway/Laerdal Global Health).<br>• Assessment of the completion and quality of data collected on birthweight at health facilities (London School of Hygiene and Tropical Medicine [LSHTM]/SNL).<br>• Evaluation of approaches to improve measurement of service	• A multi-country study (India, Malawi, Nigeria and Tanzania) is being conducted to assess the effect of continuous KMC when initiated immediately after birth compared to current practice of initiating KMC after stabilisation. The study has been ongoing since November 2017 and is scheduled to end in July 2020.<br>• In Balaka, an assessment on family compliance with continuing KMC at home following discharge generated evidence to help families to continue KMC at home.<br>• The study *Starting the conversation: community perspectives on preterm birth and kangaroo mother care in southern Malawi* found that KMC mothers and fathers only learned about KMC and care for preterm newborns after delivery of a child in need of this care.[13]<br>The series of studies about KMC implementation in Malawi has been completed. The findings were

		readiness for small and sick newborns (LSHTM/SNL).<br>• Malawi is a site in the Immediate Parent-Infant Skin-to-Skin study (IPISTOSS) looking at initiating KMC in unstable babies.	presented at the SNL KMC conference:<br>• A study about improving uptake using customised KMC wraps found that among those who used the customised wraps in skin-to-skin practices in facility KMC improved. Some 44% of mothers using a customised wrap reported 20 or more hours per day compared to 33% of mothers using the traditional chitenje.[14]<br>• A study on early outcomes among newborns discharged from KMC showed that follow-up visits averaged 88% but varied by site. One of the barriers to follow-up compliance was the distance to the facility according to mothers enrolled in the study.[15]<br>• Regarding data availability, a simplified KMC register and reporting form was developed followed by a quality assurance exercise. Annual data reporting improved with 87% of hospitals submitting KMC reports compared to 51% hospital submissions in 2014. However, data quality issues persist.[16]<br>• A study about improving quality of newborn care at a district hospital found that a reduction of 6% in neonatal death rate between 2015 (15.5%) and 2016 (9.5%) was observed after making improvements to quality of care

			which included mentorship of service providers, availability of equipment and supplies, increase number of beds in the newborn care unit from three to 40, and monthly data audits.[17]
**Knowledge Management**			
Centers of excellence or state-of-the-art facilities for KMC/care of LBW babies		Two health facilities considered KMC centers of excellence: Queen Elizabeth Central Hospital and Thyolo District Hospital. Lessons learned from these centers include that leadership should promote KMC as a priority, staff should be identified at the health facility to be trained on KMC, and improvement should be showcased when providers report and document the progress of KMC babies.	Queen Elizabeth Central Hospital and Thyolo District Hospital continue to be the two KMC centers of excellence in the country.
KMC manuals, trainings and campaigns	Between 2008 and 2011, KMC was included in maternal and newborn manuals and trainings (SN and MCHIP).	• The Care of Infants and Neonatal (COIN) course was developed by the Paediatric and Child Health Association (PACHA) of Malawi in partnership with the MOH and UNICEF in 2015 to train healthcare workers in facilities to care for young infants and newborns. This course includes a section about the three categories of KMC—facility, ambulatory and community—that exist in Malawi.[18]<br>• A task force was formed to harmonise all mentorship packages for MNH, which includes KMC. The meetings are still ongoing. All partners	• Save the Children through the SNL project generated evidence and developed an evaluation report for an SBCC campaign which included community engagement and social mobilisation designed to shift social norms and care practices for preterm and LBW babies. The campaign also targeted pregnant women to ensure that KMC counselling is provided to pregnant mothers during antennal care (ANC). Some 13 months after the implementation of the SBCC campaign, an evaluation was carried out. The evaluation showed that the campaign contributed to changes in injunctive norms around the care of newborns, increasing value for LBW

		supporting health programs are in this task force as it covers all thematic areas, e.g., MNH, nutrition, HIV, malaria, child health, etc. The task force started operating in 2017 after the establishment of the Quality Management Directorate in MOH.<br>• The National Quality of Care Tool has been adapted and is being finalised by the Quality Management Directorate and partners. The plan is to disseminate the tool with the policy and roadmap in November 2017. The Quality of Care tool is very comprehensive and includes KMC.	and preterm babies, and encouraging social support.<br>• A project that focused on strengthening facility-community linkage in care for small babies, the Every Preemie Scale, was implemented as a family-led care model in six health facilities of one district. Activities included community awareness on care for preterm/LBW babies at home. Two manuscripts are being developed: one about the caregiver/client study, and the second on the healthcare provider study.[19]
**Monitoring and Evaluation**			
KMC indicators included in the national HMIS	Thirty-two data elements were collected, but standard indicators were not defined in the DHIS2.	The DHIS2 monthly reporting forms were revised, and eight data elements and five core KMC indicators were included in 2015.	The KMC indicators currently being monitored are:<br>• KMC initiation rate: number of babies initiated in KMC (inpatient and/or ambulatory) per (i)100 livebirths at health facility and (ii) 100 LBW/premature babies identified at health facility.<br>• KMC referral completion: Proportion of babies who were initiated in KMC, referred and completed referral, and were initiated in facility based KMC.<br>• Survival to discharge: Proportion of babies initiated in facility-based KMC who are discharged alive.

			• Death before discharge: Proportion of babies initiated in facility-based KMC who died before discharge.<br>• Left against medical advice: Proportion of babies initiated in facility-based KMC who left against medical advice or abscondment.
KMC data recorded at health facilities	Some facilities used a KMC register developed by the MOH, Save the Children and partners. KMC has been part of the integrated supervision at national, zonal and district levels. KMC registers were used by some health facilities receiving partner support to track KMC services. The 2014 EmONC was the first survey to capture information about KMC services at the national level.	• In 2015, a national routine reporting system for KMC services was rolled out to replace the original KMC register and monthly report. This reporting system, comprising a register and a monthly report, tracks KMC services at the facility and district levels. Facilities report on six data elements with inpatient KMC, two data elements without inpatient KMC, and five core indicators.[20] According to an analysis of the DHIS2/health management information system 2016 data, 87% of hospitals reported providing KMC services and 45% of health facilities submitted reports on KMC. However, at the health center level, it is difficult to estimate how many facilities have operational KMC services.<br>• In 2016, an integrated neonatal register with accompanying mobile app was piloted in 10 districts, led by PACHA.<br>• The critical care pathway (bedside patient chart) for sick newborns	• Most health facilities do not consistently report through DHIS2, which makes it challenging to understand in real time whether KMC services are offered or not.<br>• At a low scale, some districts have been supported to conduct QI audits and analyse data from the KMC registers to inform program change. Currently, data collected through the DHIS2 system is only used by projects like SNL to analyse the scale of implementation. No data audits have been done at the national level to inform program changes nationwide.<br>• The sick newborn register was scaled up to all hospitals, government, CHAM and private facilities that offer nursery services. However, the roll out of the register depended on resources; hence, not all districts have received orientation.

		and a feeding log were introduced in six hospitals.	
**Advocacy**			
Professional organizations that endorse KMC	The Nurses and Midwives Council of Malawi was instrumental in including KMC in the Registered Nurse Midwifery curriculum in 2005.	PACHA advocated for KMC by introducing the COIN training course, which integrates essential newborn care and LBW baby care.[9] As of 2016, PACHA was providing KMC mentorship in ten district hospitals.	
Champions	Dr. Queen Dube, paediatrician at the Malawi College of Medicine, attended the Istanbul Convening for KMC Acceleration and was an early KMC champion in Malawi.	• There are strong local champions who promote KMC, one of them being the Chief of Health Services. There is a strong presence at the national level for KMC but a lack of resources.<br>• Dr. Queen Dube has mentored three national-level paediatric and midwife mentors who help her to provide mentorship, coaching and supervision to district hospitals for newborns.	

Table 2. Demographic and Health Survey (DHS) proxy indicators for kangaroo mother care (Malawi DHS 2015-16)[21]

**Identification of LBW Babies**	**Characteristic**	**Percentage**
Percentage distribution of live births in the 3 years preceding the survey by mother's estimate of baby's size at birth, according to background characteristics	*Very small*	4.3
	Smaller than average	11.6
Percentage of births that have a reported birth weight		83.9
Percentage of babies weighing less than 2.5 kg among births with a reported birth weight		12.3
**Initial Breastfeeding**		**Percentage**
Percentage of children born in the past 2 years who started breastfeeding within one hour of birth		76.3
Percentage of children born in the past 2 years who started breastfeeding within one day of birth		96.2

Skin-to-Skin Contact	Percentage
Percentage of births that had skin-to-skin contact for the most recent live birth in the 3 years preceding the survey	67.4

CHALLENGES

- KMC has been looked at as an additional service, which might explain why only a small percentage of preterm/LBW babies are reached with KMC services.
- Although midwives initiate preterm/LWB babies in KMC, this is not the case at some health facilities, where other services are prioritized.
- There is a need to invest funds in making KMC spaces larger, creating neonatal units of care, and increasing quality of care of preterm/LBW babies.
- KMC is taught in hospitals, which is helping to change people's attitudes, but social and behaviour change communication and advocacy for newborns needs strengthening.

LESSONS LEARNED

- Integrating KMC in national policies, guidelines and training manuals has facilitated the scale-up of KMC in Malawi.
- Local champions, including the MOH, with a track record of promoting KMC, play an influential role in scale-up and improving quality of newborn care.
- Improving health management information system data quality for KMC services will allow Malawi to identify gaps in coverage, quantify achievements, and mobilise resources for KMC better.
- The commitment of the MOH, partners and other stakeholders is critical for KMC to increase coverage and be sustainable.

FUTURE ACTIONS

- Integrate the care of small and sick babies in newborn care units in all hospitals.
- Disseminate the quality of care assessment tool and develop national quality of care standards.
- Continue the training of mentors.
- Standardise the follow-up of KMC babies after discharge.
- Allocate resources for forums to share lessons learned and for the advocacy of KMC.
- Engage private hospitals and healthcare providers in the use of KMC as an evidence-based intervention that saves lives.

DOCUMENTS AND RESOURCES

Document Title	Link to Document
Care of the Infant and Newborn in Malawi: The COIN Course Participants Manual (2015)	http://cms.medcol.mw/cms_uploaded_resources/41905_12.pdf
Social and behaviour change communication campaign *Khanda ndi Mphatso Lipatseni Mwayi* (A Baby is a Gift, give it a chance) (2016)	https://www.healthynewbornnetwork.org/hnn-content/uploads/Final-KMC-Flipchart-March-10-2016.pdf

Evaluation of Kangaroo Mother Care Services in Malawi (2012)	http://www.mchip.net/sites/default/files/Malawi%20KMC%20Report.PDF
Malawi Emergency Obstetric and Newborn Care Needs Assessment (2014)	https://www.healthynewbornnetwork.org/hnn-content/uploads/Malawi-EmONC-Report-June-2015_FINAL.pdf
Development of a National Routine Reporting System for Kangaroo Mother Care (KMC) Services in Malawian Health Facilities (2015)	https://www.healthynewbornnetwork.org/hnn-content/uploads/KMC-Register-Brief-and-Forms-Final-2015.10.09-web.pdf
Born too small: who survives in the public hospitals in Lilongwe, Malawi? (2015)	http://fn.bmj.com/content/100/2/F150
Investigating preterm care at the facility level: stakeholder qualitative study in central and southern Malawi (2016)	https://doi.org/10.1007/s10995-016-1942-z
Every Preemie Scale: Family-Led Care Model in Malawi	https://www.everypreemie.org/malawi/

REFERENCES

1. Malawi Ministry of Health (MOH). *Malawi National Guidelines for Kangaroo Mother Care*. Lilongwe: Malawi MOH; c2009.
2. Malawi MOH. *Every Newborn Action Plan: An Action Plan to End Preventable Deaths in Malawi*. Lilongwe: Malawi MOH; c2015.
3. World Health Organization (WHO). *Reaching the Every Newborn National 2020 Milestones 2017: Country Progress, Plans and Moving Forward*. Geneva: WHO; c2017.
4. Malawi Ministry of Health (MOH). Road Map for Accelerating the Reduction of Maternal and Newborn Mortality and Morbidity in Malawi. Lilongwe: Malawi MOH; c2005.
5. Malawi MOH. *Malawi Child Health Strategy 2014–2020*. Lilongwe: Malawi MOH; c2013.
6. Malawi MOH and United Nations Population Fund (UNFPA). National Sexual and Reproductive Health and Rights (SRHR) Policy (2017-20022). Lilongwe: Malawi MOH; c2017. Available from: https://malawi.unfpa.org/sites/default/files/resource-pdf/Malawi_National_SRHR_Policy_2017-2022_16Nov17.pdf.

7.Malawi MOH. *Malawi National Reproductive Health Service Delivery Guidelines 2014–2019*. Lilongwe: Malawi MOH; c2014.

8.Chavula K, Likomwa D, Guenther T, et al. Readiness of hospitals to provide kangaroo mother care (KMC) and documentation of KMC service delivery: analysis of Malawi 2014 emergency obstetric and newborn care (EmONC) survey data. *J Glob Health*. 2017;7(2):020802. doi:10.7189/jogh.07.020802.

9. Saving Newborn Lives (SNL). *Measuring strength of implementation and effective coverage of Kangaroo Mother Care intervention in Malawi*. Presented at: SNL Legacy Webinar October1, 2020. Available from: https://www.healthynewbornnetwork.org/hnn-content/uploads/KC-_Pathway-to-effective-coverage_SOI_MW-_290920.pdf.

10.Bergh A, Banda L, Lipato T, Ngwira G, Luhanga R, Ligowe R. *Evaluation of Kangaroo Mother Care Services in Malawi*. Fairfield, CT: Save the Children; c2012.

11. Ahlsén AK, Spong E, Kafumba N, Kamwendo F, Wolff K. Born too small: who survives in the public hospitals in Lilongwe, Malawi? *Arch Dis Child Fetal Neonatal Ed*. 2015;100(2):F150–F154. doi:10.1136/archdischild-2013-305877.

12. Gondwe A, Munthali A, Ashorn P, Ashorn U. Investigating preterm care at the facility level: stakeholder qualitative study in central and southern Malawi. *Matern Child Health J*. 2016;20(7):1441–1447. doi:10.1007/s10995-016-1942-z.
13. Lydon M, Longwe M, Likomwa D, et al. Starting the conversation: Community perspectives on preterm birth and kangaroo mother care in southern Malawi. *J Glob Health*. 2018;8(1). doi:10.7189/jogh.08.010703
14. Chavula K, Guenther T, Valsangkar B, et al. Improving skin-to-skin practice for babies in kangaroo mother care in Malawi through the use of a customized baby wrap: A randomized control trial. *PLoS One*. 2020;15(3):1-16. doi:10.1371/journal.pone.0229720
15. Dube Q. Kangaroo Mother Care (KMC) Follow *Up Study: Early outcomes among newborns discharged from facility based KMC in Malawi*. 29th International Pediatric Association Congress to be held in Panama City, Panama. Available from: https://resourcecentre.savethechildren.net/library/kangaroo-mother-care-kmc-follow-study-early-outcomes-among-newborns-discharged-facility.
16. Chavula K. Improving Availability, Quality, and Use of Routine date for Newborns: Malawi's experience with kangaroo mother care. 29th International Pediatric Association Congress to be held in Panama City, Panama. Available from: https://resourcecentre.savethechildren.net/library/improving-availability-quality-and-use-routine-date-newborns-malawis-experience-kangaroo.
17. Dube Q, Chavula K, Guenther T, et al. Improving Quality of Newborn Care with a Structured Mentorship Approach at a District Hospital in Malawi. Available from: https://fundacioncanguro.co/wp-content/uploads/2018/11/What-does-it-take-to-scale-up-KMC.-Lessons-from-Malawi.pdf.
18. Malawi MOH, Paediatrics and Child Health Association. *Care of the Infant and Newborn in Malawi: The COIN Course Participants Manual*. Lilongwe: Malawi MOH; c2015.
19.Every Preemie Scale Malawi. Available from: https://www.everypreemie.org/malawi/.
20.Save the Children. *Development of a National Routine Reporting System for Kangaroo Mother Care (KMC) Services in Malawian Health Facilities*. Lilongwe: Save the Children Malawi; c2015.
21.Malawi National Statistical Office (NSO) and ICF. *Malawi Demographic and Health Survey 2015–16*. Zomba: NSO and ICF; c2017.

KANGAROO MOTHER CARE IN RWANDA

OVERVIEW

The Ministry of Health (MOH) has committed to improving the health outcomes of preterm and low birth weight (LBW) babies by leading the scale-up of facility-based KMC services in collaboration with partners and stakeholders since 2007. KMC is provided in all district hospitals. KMC has been included in the national *Maternal, Newborn and Child Health (MNCH) Strategic Plan for 2018-2024*[1] and in the national guidelines for care of small babies, including the *Neonatal Protocol* Manual and the *Essential Newborn Care Reference Manual*.[2] The *Neonatal Protocols* manual outlines that healthcare providers should encourage all mothers of stable LBW babies (< 2,000 g) to provide KMC to prevent hypothermia, enable frequent breastfeeding, and allow for an earlier hospital discharge. The *Essential Newborn Care Reference Manual* describes KMC along with its advantages, its discharge criteria, and the importance of follow-up within 1 week of discharge from the district hospital.[3] As the country transitions from the scale-up of KMC services to sustainability and quality, there are opportunities for improvement regarding the roll-out of follow-up after discharge and improving data quality.

Table 1. Status of KMC in Rwanda by strategic area

Domain	Prior to and during 2014	2015-2017	2017-2019
**Policy**			
National Health Policy	Facility-based kangaroo mother care (KMC) was included in the National Child Health Policy in April 2009.	The Rwanda MOH is currently reviewing an RMNCH policy that includes KMC. It is expected that the MOH will sign and approve the policy later in 2017.	The Rwanda MOH has included KMC as part of the MNCH strategic plan 2018-2024 in the list of MNCH package of care priority areas at all levels (referral, district, health center (HC), and community) for newborns.[1]
National Guidelines	• KMC was integrated in the *Neonatology Clinical Treatment Guidelines*,[4] and in the *National Neonatal Care Protocol*.[3]<br>• National KMC guidelines were developed and published in November 2011.	• KMC guidelines are being updated with greater detail.<br>• KMC is part of the *Essential Newborn Care Guidelines*; the most recent version was published in December 2015.[2]	Guideline documents that include KMC as a standard of care are:<br>• *MNCH strategic plan*.<br>• *Essential Newborn Care*.<br>• *The National Neonatal Care Protocol* was updated in 2019 and is currently being reviewed for approval.
**Country Support/Implementation**			
Levels and types of facilities implementing KMC	KMC was introduced as a pilot in Muhima District Hospital in Kigali in 2007. Subsequently, KMC was scaled to eight district-level facilities from 2007 to 2010.[5] According to an assessment	It is recommended in Rwanda's *Neonatal Protocols* that there should be at least four to six beds in each district hospital designated for KMC. LBW babies born in first-	KMC is implemented in tertiary, teaching, provincial and district hospitals. Some HCs have also started conducting ambulatory KMC for follow-up as part of the paediatric development clinic program. USAID through

	conducted in 2012, 30 of 40 sampled district hospitals had some KMC services.[6]	level facilities are referred to district hospitals, where they are stabilised and initiated in KMC.[3]	the Ingobyi Activity (Intrahealth) is planning to support the piloting of follow-up KMC services at HCs in one district to scale it later to other districts.
Percentage of LBW newborns initiated in facility-based KMC		An estimated 75% or more LBW newborns are initiated in facility-based KMC, as KMC is part of the national protocol. District hospitals are implementing KMC, but data are needed to support this estimate.	An estimated of 80% LBW newborns are initiated in facility-based KMC. This estimate is based on HMIS data from 20 districts from October 2018-September 2019.
Funding	The national health insurance scheme, Mutuelle Sante (used by 90% of Rwandans) includes KMC in its coverage.	Because KMC is under the RMNCH umbrella and KMC is integrated in newborn care, KMC funding mostly comes from MOH funds.	KMC funding is a combination of MOH and donor funds.
**Research**			
Major or program-based studies currently being conducted related to KMC		• A study was conducted in the Kabutare District Hospital about KMC follow-up after discharge.[7]<br>• A health partnership to reduce neonatal mortality in four hospitals in Rwanda noted that in a large district hospital 42 mothers provided KMC in the year before the program, which increased to 71/year followed by 157/year during the program. [8]	A learning agenda on the follow-up of babies at risk in HC is being developed.
**Knowledge Management**			
Centers of excellence or state-of-the-art facilities for	The Muhima District Hospital was established as center of excellence in August 2007. This initiative was led by the MOH with support from the ACCESS project.		

KMC/care of LBW babies			
KMC manuals, trainings and campaigns	• KMC was integrated in the basic reference manual of emergency obstetrics and newborn care in 2009, in the *Essential Newborn Care Reference Manual* in 2011, and in maternal community health worker trainings.		
**Monitoring and Evaluation**			
KMC indicators included in the national HMIS	KMC indicators were integrated in HMIS in 2013. KMC admission and KMC follow-up in district hospitals were disaggregated by preterm and LBW babies.	KMC indicators continue to be included in the HIMS. However, the denominator of the KMC indicator includes all newborn babies. Currently, there are discussions about changing the denominator to babies who weigh 2 kilos or less.	• The denominator for the KMC indicator has been changed to babies who weigh 2 kilos or less.<br>• The indicator to track follow-up visits has been added in the neonatal and KMC registers.
KMC data recorded at health facilities	KMC was not documented in a standard way prior to 2013.	Health facilities providing KMC services track service delivery using the KMC register. Facilities then submit monthly reports on KMC indicators that are included in the HMIS. There are opportunities for improvement of data quality and completeness.	• The neonatal and KMC registers now include information to track up to three KMC follow-up visits.
**Advocacy**			
Professional organizations that endorse KMC	There were two main professional organizations endorsing KMC: the Rwanda Paediatric Association and the Rwanda Association of Midwives.	The Rwanda Paediatric Association and the Rwanda Association of Midwives have endorsed KMC; both organizations promote KMC through trainings and mentorship in health facilities.	
Champions	There were champions at different levels such as the MOH, partners and healthcare facilities advocating for	There are strong local KMC champions but there is a lack of resources for monitoring and	Champions advocated for the inclusion of KMC indicators in the HMIS, availability of

	KMC. Some of the champions were Dr. Agnes Binagwaho, former Minister of Health, and Dr. Mwali (Assumpta) Kayinamura.	follow-up. The MOH continues to be a strong champion given its commitment to and continuous engagement with KMC.	KMC units in all facilities, and the referral of all babies to KMC positions.

Table 2. Demographic and Health Survey (DHS) Proxy Indicators for kangaroo mother care

DHS Indicators Related to KMC (Rwanda DHS, 2014-15)[9]		
**Identification of LBW babies**	**Characteristic**	**Percent**
Percentage distribution of live births in the 3 years preceding the survey by mother's estimate of baby's size at birth, according to background characteristics	*Very small*	3
	Smaller than average	12.9
Percentage of births that have a reported birth weight		92.2
Percentage of babies weighing less than 2.5 kg among births with a reported birth weight		6.3
**Initial Breastfeeding**		**Percent**
Percentage of children born in the past 2 years who started breastfeeding within one hour of birth		80.5
Percentage of children born in the past 2 years who started breastfeeding within one day of birth		95.7
**Skin-to-Skin Contact**		**Percent**
Percentage of births that had skin-to-skin contact for the most recent live birth in the 3 years preceding the survey		N/A

CHALLENGES

- There are major funding gaps for KMC, a need for more beds and space for KMC in some hospitals, lack of food at hospitals for new mothers and a lack of formula for LBW babies.
- It is not always possible for mothers to bring the babies back to health facilities for check-ups due to long distances and lack of access to transportation.
- There is a shortage of staff to conduct monitoring during hospitalisation.

LESSONS LEARNED

- The strong leadership from the MOH and the support of partners and other stakeholders have been essential to the scale up of KMC. KMC has been integrated into national policies and guidelines, as well as in the HMIS and registers.
- WhatsApp is used in some districts to follow up with mothers of LBW babies.
- KMC messages are disseminated via radio, community gatherings and theatrical presentations.

FUTURE ACTIONS

- Continue training health providers because there is a high turnover of personnel.

- Offer mentorship on MEN approaches and complete documentation of feeds (nutrition methods, types, frequency) on existing national newborn medical record forms.
- Develop guidelines and a clear plan to follow up preterm/LBW babies after discharge until they reach 1 year of age.
- Engage leaders, such as the minister of health, the director of affairs, and the directors of hospitals, to continue advocating for the acceleration of KMC.
- Include quality improvement activities focused on family-centered care (ongoing promotion of companion of choice, intermittent KMC, engagement of family members in KMC and improved counselling).
- Integrate KMC into the nursing curriculums.
- Engage private practitioners and private clinics.

DOCUMENTS AND RESOURCES

Document Title	Link to Document
Neonatal Protocols (2011)	http://www.childrenshospital.org/~/media/research-and-innovation/divisions/newborn-medicine/neonatalprotocolsrwanda.ashx
Neonatology Clinical Treatment Guidelines (2012)	http://www.moh.gov.rw/fileadmin/templates/Norms/Neonatology-Clinical-Treatment-Guidelines-OTHER-VERSION.pdf
Evaluation of Kangaroo Mother Care Services in Rwanda (2012)	http://reprolineplus.org/system/files/resources/Rwanda%20KMC%20Evaluation%20Report_0.pdf
Early outcomes of preterm babies hospitalized in kangaroo mother care units in Rwanda (2014)	http://fundacioncanguro.co/es/documentacion/docuementos/doc_download/364-13-early-outcomes-ppt-assumpta-mwali-rwanda.html
Follow-up assessment of preterm infants in southern Rwanda: final results (2015)	http://www.vlaamsegezondheidszorg.com/sites/default/files/august10_ppt_for_moh_3_dec_2015.pdf
Maternal Newborn and Child Health Strategic Plan (2018–2024)	http://www.moh.gov.rw/fileadmin/Publications/Strategic_Plan/Rwanda_MNCH_StrategicPlan_June_costed_v2Draft.pdf

REFERENCES

1. Rwanda MOH. *Maternal Newborn and Child Health Strategic Plan (2018–2024)*. Kigali: Rwanda MOH; c2018.
2. Rwanda MOH. *Essential Newborn Care Reference Manual*. Kigali: Rwanda MOH; c2015.
3. Rwanda MOH. *Neonatal Protocols*. 2nd ed. Kigali: Rwanda MOH; c2014.
4. Rwanda MOH. *Neonatology Clinical Treatment Guidelines*. Kigali: Rwanda MOH; c2012.
5. Kayinamura M. Early outcomes of preterm babies hospitalized in kangaroo mother care units in Rwanda. Presented at: the 10th International Conference on Kangaroo Mother Care, Kigali, Rwanda, November 17–19, 2014.
6. Bergh A, Sayinzoga F, Mukarugwiro B, Zoungrana J, Abayisenga G, Karera C, Ikiriza C. *Evaluation of Kangaroo Mother Care Services in Rwanda*. Kigali: Rwanda Ministry of Health (MOH); c2012.

7. Cannoodt L, Kayinamura M. Follow-up assessment of preterm infants in southern Rwanda: final results. Presented at: Rwanda MOH, Kigali, December 3, 2015.
8. Ntigurirwa P, Mellor K, Langer D, et al. A health partnership to reduce neonatal mortality in four hospitals in Rwanda. *Globalization and Health*. 2017;13:28. doi:10.1186/s12992-017-0252-6.
9. National Institute of Statistics of Rwanda (NISR), Ministry of Health Rwanda, and ICF. *Rwanda Demographic and Health Survey 2015–16*. Rockville, Maryland: USA; c2016.

Tables S4

Status of facility-based KMC activities in other countries of interest and the Dominican Republic

Eight countries of interest and the Dominican Republic participated in the assessments. Participants from four countries (Mali, Mozambique, Pakistan and the Philippines) provided information in the 2014, 2017 and 2019 assessments. Participants from China provided information for the 2017 and 2019 assessments, and participants from Uganda, Vietnam and Tanzania provided information in 2014 and 2017. Participants from the Dominican Republic provided information in the 2019 assessment.

**Status of facility-based KMC activities in China**<br>Participants provided information in 2017 and 2019.
**Policy**<br>• In 2015, delegates from the MOH, MCH center, hospitals, and Save the Children China visited Save the Children UK to observe KMC initiation for stable newborns.<br>• The London School of Hygiene and Tropical Medicine conducted a scoping visit to China to four hospitals, including three tertiary hospitals and one rural hospital.<br>• In 2016, delegates from China's key MCH center and hospital and Save the Children China visited Washington, DC to discuss the KAP, KMC research and lessons learned from other countries.<br>• A standardised policy to provide KMC for small babies is being developed. The National Health Commission (NHC) is expected to release the policy in 2020.<br>• In 2017, senior delegates from MOH, key pilot hospitals and Save the Children China visited Bogota, Colombia to witness its KMC model.<br>• The national guidelines are being piloted as part of the implementation research in 21 hospitals. The guidelines will be released in mid-2020.<br>**Country Support/Implementation**<br>• KMC implementation research started in 2017, in eight hospitals.<br>• In the eight hospitals where KMC was initially piloted, about 20% of small babies were initiated in KMC in 2017. The low initiation might be due to factors such as limited space and a lack of human resources.<br>• By 2019, KMC implementation research had been extended to 21 hospitals across all levels in 11 provinces.<br>• It was reported that KMC sessions varied by hospitals in 2019. Some hospitals provided 1-2-hour sessions. A few hospitals allowed some parents to provide KMC 8 hours per day.<br>• The estimated number of small babies initiated in KMC remained at 20% in 2019, possibly due to the limited space in NICUs and limited human resources.<br>**Monitoring and Evaluation**<br>• In 2019, KMC indicators were not included in the national HMIS and there was no plan for KMC indicators to be included. Guidelines will have optional indicators.<br>• KMC data collection tools were adopted from other countries, and KMC protocols, one for babies who are in the NICU and one for late preterm babies in postpartum rooming-in settings, are being drafted.<br>**Research**<br>• Studies with the findings from the pilot that is ongoing in 21 hospitals will be released in 2020.<br>**Knowledge Management**<br>• Five KMC Facilities have been nominated as centers of excellence:<br>o First Peking University Hospital.<br>o Xiangya Medical University Hospital.<br>o Xibei Maternal and Child Health Hospital.<br>o Hunan Provincial MCH Hospital.<br>o Nanjing MCH Hospital.<br>• Two of these nominated facilities are training two nearby hospitals in KMC:<br>o Xiangya Medical University Hospital.<br>o Xibei Maternal and Child Health Hospital.

Advocacy

- As of 2019, none of the professional organizations had endorsed KMC due to the limited evidence about KMC services in China.
- Dr. Gengli Zhao and Dr. Qi Feng are considered leading experts for improving health outcome for small babies. They both work at the 1st Peking University Hospital and have been KMC champions conducting trainings, leading the drafting of the implementation research protocols and conducting monitoring visits to other hospitals.

Challenges

- There is limited access to and space in NICUs, which affects the provision of KMC services.

Future Actions

- Finalising and obtaining approval of the KMC guidelines.
- Standardising KMC services and increasing their availability.

Status of facility-based KMC activities in the Dominican Republic

Participants provided information in 2019.

Policy

- KMC was included in the operational plan to accelerate the reduction of maternal and child deaths.[24]
- In 2018, the guidelines for the care of preterm babies were released. KMC is mentioned throughout the document as a clinical treatment that is safe and effective for the care of preterm newborns in all hospitals. One of the recommendations is that if the newborn's temperature is stable and there is an appropriate gain weight during KMC, it is advised to continue KMC for the maximum time possible. The guidelines also recommended the continuation of KMC after discharge.[25]
- The Ministry of Public Health in collaboration with the MOH, KMC advocates, Project Hope, and the Dominican Kids foundation drafted a protocol for KMC, based on the guidelines provided by the Fundación Canguro in Bogotá, Colombia. The protocol will be reviewed and validated by Dr. Nieves Rodríguez, Dr. Nathalie Charpak and Dr. Goldy Mazia.

Country Support/Implementation

- KMC was introduced in the Dominican Republic in 2009 through the USAID-funded project MCHIP at the regional Hospital San Vicente de Paul, which in 2012 became a KMC center of excellence and training institution.
- Seven hospitals provide KMC services—five regional hospitals and two national referral hospitals. These hospitals cover five out of nine health geographical regions. In 2020, it is projected that KMC will be expanded to five hospitals in three more health geographical regions. The MOH is leading this initiative with the support of UNICEF, Project Hope and Dominican Kids.
- There are no statistics available to estimate the percentage of newborns who are initiated in KMC.
- It is routine to place newborns in skin-to-skin position in private and public hospitals of all levels. Even in referral hospitals, the MOH advises that newborns are transported in KMC position to prevent hypothermia.
- KMC is funded through donors and the MOH.

Monitoring and Evaluation

- KMC is not tracked through the HMIS, but there are plans to include KMC indicators.
- Hospitals track KMC implementation through registries, but KMC indicators have not been included in national statistics.

Knowledge Management

- KMC is being included in the nursing curriculum at the Universidad Autónoma de Santo Domingo.
- Neonatologists who do rotations through the Los Mina hospital are trained on KMC and most of them implement KMC in their workplaces, either public or private.
- There is a high demand for KMC services among mothers.
- There has been collaboration for best practices through the Fundación Canguro in Bogotá, Colombia which has conducted evaluations in all hospitals that offer KMC services.

Advocacy

- There are SBCC campaigns to promote KMC via radio, TV and newspapers.

Community Engagement

- There are private clinics that refer LBW and/or preterm babies to the hospitals with KMC programs.
- The Sociedad de Medicina Prenatal is among the professional organizations that endorse KMC.

Challenges

- There is lack of human resources, which could be addressed by training new personnel and reorganising existing personnel according to the KMC guidelines.
- More funding is needed to develop job aids and to provide kangaroo wraps.

- There is a need to improve data collection, data quality and data use while strengthening ambulatory KMC follow-up.

Future Actions

- Validate and launch the KMC protocols.
- Ensure the sustainability of the KMC program.
- Continue documenting lessons learned and progress achieved on reducing neonatal survival by implementing KMC, as this was key to getting buy-in from hospital directors and the MOH.

Status of facility-based KMC activities in Mali

Participants provided information in 2014, 2017 and 2019.

Policy

- The national action plan for newborn health includes scaling up KMC with the financial support of partner organizations. Since 2006, KMC has been included in the Policy Norms and Procedures (PNP/SR) in the strategic plan for reproductive health, which was updated in 2018.
- Mali developed a specific plan for newborns that includes the extension of KMC to community health centers (CScom), the first primary care facilities. KMC is included in the training materials for essential newborn care for health providers and in the training materials for community health workers (CHWs), the latter focusing on referral and follow-up of ambulatory KMC.
- KMC activities are funded through a combination of donor and MOH monies.

Country Support/Implementation

- KMC used to be implemented only at the hospital level, but with the support of partner organizations, KMC has been scaled up and is now practiced in some health facilities at all levels. As of December 2019, there were 461 sites in USAID-funded *Services de Santé à Grand Impact* (SSGI)-supported districts trained in KMC services (30 referral district hospitals and 431 community health centers).
- According to the HMIS, in 2018, 30% of preterm/LBW newborns were initiated in facility-based KMC in the SSGI-supported region. In 2019, at the 461 USAID/SSGI-supported health facilities, it was estimated through the HMIS that 46% of babies weighing < 2,500 g were initiated in facility-based KMC (DHIS2).

Region/Metric	LBW 2018	LBW 2019	LBW Trends	KMC 2018	KMC 2019	KMC Trends	% Increase KMC
Kayes	4,645	4,054		1,961	2,687		37%
Sikasso	7,995	6,631		2,177	2,686		23%
Koulikoro	5,887	5,996		1,505	2,317		54%
**Total**	**18,527**	**16,681**		**5,643**	**7,690**		**36%**

Number of LBW newborns and KMC initiations by year and region

- It is estimated that over 75% of newborns are placed in immediate skin-to-skin contact after delivery and prior to cutting the umbilical cord. After the cord is cut and the baby is dried, the baby is returned to the mother's chest. The newborn health national guidelines recommend skin-to-skin contact as a practice that should be initiated at birth.

Monitoring and Evaluation

- KMC services are tracked via the National Health Information System, DHIS2, which includes two KMC indicators: (i) number of LBW babies born < 2,500 g and (ii) number of LBW babies initiated in KMC.

Knowledge Management

- The Gabriel Toure University Teaching Hospital (CHU-Gabriel Toure) is a health facility considered as a center of excellence for KMC. There, KMC is taught as standard of care for managing preterm/LBW babies.

Advocacy

- The Malian Paediatrics Association (*Association Malien de Pediatrie* [AMAPED]) endorses KMC by supporting KMC trainings and supervision and by promoting KMC specially during World Prematurity Day.

Community Engagement

- As part of maternal and newborn health activities, Community Health Workers (CHWs) raise awareness on KMC and follow-up on babies on KMC. CHWs sensitise community members on newborn danger signs. They also do postnatal visits within 24 hours of birth, and on Day 2, Day 5, between Days 7 and 14 and between Days 15 and 28.

Challenges

- Lack of space at the health facilities for in-patient care, lack of resources (for example, food or transportation) to support mothers who need in-patient or ambulatory care, limited job aids and tools and the need to provide more trainings and refreshers for providers to ensure sustainability.

Future Actions

- Continuous reinforcement of KMC skills especially in basic emergency obstetric and newborn care (BEmONC) facilities and systematic integration of KMC in maternal and newborn health services.
- National policies, norms and procedures on KMC need to provide specific directives for all health system levels (national, regional, district hospitals and community health centers).
- The KMC register needs to be validated nationally and integrated in national forms/registers. The register should include KMC follow-up information.

Status of facility-based KMC activities in Mozambique

Participants provided information in 2014, 2017 and 2019.

Policy

- KMC is included in strategic documents on child health including essential care for newborns and in packages of continuous training for maternal and neonatal care.
- The newborn care action plan (ENAP) has been approved. The ENAP includes guidance on KMC.

Country Support/Implementation

- KMC implementation is low (10-< 25%) given that most births happen at the health facilities in the lowest tier, which lack the infrastructure to care for preterm/LBW babies.

Monitoring and Evaluation

- KMC indicators are not yet included in the national HMIS and the percent of premature newborns who receive KMC is only tracked at the program level.
- Standardised KMC registries are being developed.
- The Ministry of Health (MISAU) periodically collects information on KMC at the health facility during evaluation activities.

Knowledge Management

- A pocket-book for the care of hospitalised newborns will be finalised in early 2020.

Advocacy

- The Associação Moçambicana de Pediatras (AMOPE) is one of the professional organizations that endorse KMC. Supported by UNICEF, AMOPE has been involved in trainings about caring for preterm and sick babies in three provinces, and it is collaborating in the development of the pocket-book about caring for sick newborns.

Challenges

- Although KMC must be practiced in all health facilities (public or private) the lack of space, mostly in primary healthcare facilities, limits KMC implementation.

Future Actions

- To scale up KMC, it is critical that the MOH with the support of partners and professional associations conducts trainings, increases the availability of human resources, develops materials, purchases equipment and designates spaces for KMC.

Status of facility-based KMC activities in Pakistan

Participants contacted in 2014, 2017 and 2019

Policy

- KMC has been integrated in policies such as the RMNCH, the Every Newborn Action Plan, and the IMNCI. Through the USAID's Maternal and Child Health Integrated Program (MCHIP), KMC units were established along with manuals for health providers and community health workers. Recently, the National Health Ministry in collaboration with the World Health Organization and partners has developed policy guidelines for facility-based KMC.
- There is no uniform policy for KMC implementation, and as of today, all partners implement and monitor KMC activities separately.

Country Support/Implementation

- KMC is relatively new in Pakistan. MCHIP through Save the Children started implementing KMC as a pilot in 2017 in one hospital: the Gambat Institute of Medical Sciences (GIMS) in Khairpur. Later, the District Headquarters Hospital in Shikarpur started offering KMC services, but due to lack of funds, the program closed.
- Currently, KMC is practiced in two government hospitals: GIMS, Khairpur and the Jacobabad Institute of Medical Sciences (JIMS) in Jacobabad district. This implementation is led by the Department of Health (DOH) with support from Save the Children.
- KMC funding is a combination of donor and MOH funding.

Monitoring and Evaluation

- There is no system in place to track availability of facility-based KMC services.

- KMC indicators have not been included in the national HMIS.

Knowledge Management

- A qualitative study to identify barriers and enablers for practicing KMC in rural Sindh was conducted. Two hospitals were included in the study: the CEmONC hospital GIMS in Khairpur district, and the District Headquarters Hospital in Shikarpur. The key facilitators for practicing KMC were ownership of hospital management, close coordination between the obstetrics and paediatrics departments, involvement of community health workers, strong linkages between hospital and community health workers, presence of a counsellor and trained staff in KMC units, and ensuring privacy, food and security for female attendants. Lack of government support, unwillingness of hospital staff, poor follow-up processes after discharge and weak data management and analysis are few of the barriers listed in the study.[26]

Advocacy

- Save the Children Pakistan developed a short film on KMC implementation experience, which is widely accepted in the country.

Challenges

- Lack of funding.

Future Actions

- Political commitment will be essential in the upcoming years to scale up KMC.
- There is a high level of acceptance of KMC within government and community; unfortunately, there is a lack of funds either to continue KMC in the two existing hospitals or to plan for further scale-up in other districts.

Status of facility-based KMC activities in the Philippines

Participants provided information in 2014, 2017 and 2019.

Policy

- Facility-based KMC is part of the national policy endorsed by the Department of Health (DOH) in its Administrative Order 2009-2025 "Essential newborn care: adopting policies and guidelines on essential newborn care."
- KMC initiation and ambulatory KMC are recommended services for small babies listed in the Republic Act 11148 – *Kalusugan at Nutrisyon ng Mag-Nanay* Act (Health and Nutrition of Mother and Child), which was passed into law in 2018.
- The DOH is drafting a "National policy on the quality of care for small babies: accelerating the reduction of newborn deaths," which focuses on small babies. Training of healthcare providers focuses on the prevention of the complications of prematurity and LBW, the importance of early initiation of KMC at birth, and its continuation.
- The DOH is currently updating the Philippines Newborn Action Plan 2030 to address the acceleration of improvement of quality of care for small babies.

Country Support/Implementation

- It is estimated that 58% of preterm babies and babies weighing 2,000 g or less in national hospitals, and 38% in subnational hospitals are initiated in KMC.[6]
- About 28% of small babies in national hospitals, and 48% in subnational hospitals, are initiated in immediate skin-to-skin contact.[6]
- The number of KMC service delivery points has increased significantly from 21 in 2013 to 145 in 2020. This was achieved through collaborative efforts between WHO, UNICEF, DOH, the KMC Foundation Philippines and the Association of Philippine Schools of Midwifery (APSOM).
- KMC initiation, as part of delivery room resuscitation has been incorporated into the Neonatal Resuscitation Philippines Plus (NRPh+) of the Philippine Society of Newborn Medicine and endorsed by the Philippine Paediatric Society as its official training module for paediatric residents, neonatology fellows-in-training and consultants renewing their NRP providers' status.

Monitoring and Evaluation

- A system for integrating services such as the availability of facility-based KMC services, prevention of complications of prematurity and low birthweight, basic newborn resuscitation, and community mobilisation into the monitoring system has started.
- KMC data is recorded at the health facilities through a KMC Audit Form. KMC indicators have not been included in the hospital information management system (HIMS), but there is a plan to collect the data in a more systematic way at the DOH level.

Funding

- KMC activities are funded through a combination of development partners and DOH funding, although most of the funding is from the DOH. There are gaps in funding quality improvement activities.

Knowledge Management

- A health insurance package for preterm and small babies, "Z Benefit Package for Preterm and LBW infants," was developed in collaboration between the DOH and the Philippine Health Insurance Corporation (PhilHealth) with technical assistance from UNICEF.

Challenges

- A challenge in the scale-up of KMC nationwide, has been the regional scale-up to the local government unit level because some areas are geographically isolated and disadvantaged.
- Lack of human resources for health at the subnational and local government unit level to provide technical assistance to health facilities and health workers hamper the further scale-up of KMC.

Future Actions

- The next steps are for regional centers of excellence in KMC/CSB to collaborate with the DOH to roll out KMC to all Level 2 and Level 3 hospitals.
- The DOH and PhilHealth will facilitate the implementation and availability of the national health insurance package for premature and small babies nationwide.
- Each center of excellence will integrate the KMC data base into their HIMS starting 2020

Status of facility-based KMC activities in Uganda, Vietnam, and Tanzania

Participants provided information in 2014 and 2017.

Status of facility-based KMC activities in Uganda

Initially, KMC was implemented on a small scale in Uganda. Later, SNL used KMC to increase the survival of small babies in western Uganda at the Regional Hoima Referral Hospital, where they set up a regional learning center and implemented KMC in lower level facilities. In total there are 11 facilities providing KMC. The MOH is currently reviewing how to adapt KMC indicators for inclusion in the National Newborn Care Guidelines. The SNL protocols and tools are being used in central Uganda in three districts where there are three KMC model sites in each district and KMC corners in six Level 3 facilities. Some of the challenges in implementing KMC have been the designation of KMC spaces due to limited space at the health facilities, being able to keep mothers on the ward until the baby reaches 1.5 kg, which is the discharge criterion because food is not provided for the mothers, and not having enough staff to monitor daily weight and temperature of the babies in KMC. The next steps are to finalise the KMC tools and guidelines to be scaled up.

Status of facility-based KMC activities in Vietnam

The progress achieved through partner organizations in the care of small babies served to plan for the scale-up of KMC. KMC is included in the Early Essential Newborn Care Guideline and in the National Guideline on RH services. Most tertiary, teaching, and secondary/district facilities provide KMC as well as some of the private hospitals. According to the 2016 MOH annual report, KMC services were provided in 60 of 64 provinces throughout Vietnam, and 36,708 preterm newborns received KMC services in 2016 (the total number of live children was 1,563,231). Funding for KMC activities is a combination of MOH and partner funding, but since Vietnam became nominated as a middle-income country, the funding sources to implement health projects have been significantly reduced. Currently the government is committed to scale up KMC integration with the EENC package. It is expected that Vietnam will update the KMC guidelines to include information about post-discharge follow-up services for preterm/LWB and to improve data quality in hospitals.

Status of facility-based KMC activities in Tanzania

There are KMC national guidelines and KMC is covered under newborn care services. However, KMC as a standard of care is not yet emphasised in newborn health policies. Approximately 20% of health facilities provide KMC services. The government set up a plan to scale up KMC but due to budget constraints, the scale-up has been delayed. Although KMC indicators are not included within the HMIS system, there is a separate KMC data register that is used at health facilities. One of the most significant achievements in increasing access to KMC services has been the recognition by the MOH of 11 centers of quality service provision and the setting up of KMC services in 25 hospitals in the provinces of Mara and Kagera. The next steps to scale up KMC in Tanzania will be to include KMC indicators in the HMIS system and to integrate KMC standards in the C/BEmONC as well as scaling up KMC in more health facilities with the support of partners.

REFERENCES

1. UNICEF. Levels & Trends in Child Mortality: Report 2019-Estimates developed by the UN Inter-agency Group for Child Mortality Estimation. New York: UNICEF/WHO/WB/UN*;* c2019:1-32.
2. United Nations. The Millennium Development Goals Report. *United Nations*. c2015:72. Available from: https://www.un.org/millenniumgoals/
3. United Nations. United Nations Millennium Declaration. New York: United Nations; c2000.
4. World Health Organization. *Survive and Thrive: Transforming Care for Every Small and Sick Newborn*. Geneva: WHO; c2018.
5. Chawanpaiboon S, Vogel JP, Moller AB, et al. Global, regional, and national estimates of levels of preterm birth in 2014: a systematic review and modelling analysis. *Lancet Glob Heal*. 2019;7(1):e37-e46. doi:10.1016/S2214-109X(18)30451-0
6. World Health Organization. Kangaroo mother care to reduce morbidity and mortality in low-birth-weight infants. Available from: https://www.who.int/elena/titles/kangaroo_care_infants/en/. c2019
7. Harrison MS, Goldenberg RL. Global burden of prematurity. *Semin Fetal Neonatal Med*. c2016;21(2):74-79.
8. World Health Organization. Kangaroo mother care: A practical guide. *WHO Reprod Heal Res*. c2003;73(5):1-54.
9. Lawn JE, Mwansa-Kambafwile J, Horta BL, Barros FC, Cousens S. “Kangaroo mother care” to prevent neonatal deaths due to preterm birth complications (Structured abstract). *Int J Epidemiol*. 2010;2(Supplement 1):i144-i154. doi:10.1093/ije/dyq031
10. World Health Organization. *WHO Recommendations on Interventions to Improve Preterm Birth Outcomes*. Geneva: WHO; 2015.
11. World Health Organization. Kangaroo mother care: A practical guide. *WHO Reprod Heal Res*. c2003:1-48. Available from: https://www.who.int/publications/i/item/9241590351
12. Chan GJ, Labar AS, Atun R, Chan GJ. Kangaroo mother care: a systematic review of barriers and enablers Systematic reviews. *Bull World Heal Organ*. c2016:130-141.
13. Conde-Agudelo A, Díaz-Rossello JL. Kangaroo mother care to reduce morbidity and mortality in low birthweight infants (Review) Kangaroo mother care to reduce morbidity and mortality in low birthweight infants. *Cochrane Database Syst Rev*. 2014;(4):4-6.
14. Boundy EO, Dastjerdi R, Spiegelman D, et al. Kangaroo mother care and neonatal outcomes: a meta-analysis. *Pediatrics*. c2015;131(1).
15. Charpak N, Tessier R, Ruiz JG, et al. Twenty-year follow-up of kangaroo mother care versus traditional care. *Pediatrics*. c2017;139(1).
16. World Health Organization. *Every Newborn an Action Plan to End Preventable Deaths*. Geneva: WHO; c2014.
17. Engmann C, Wall S, Darmstadt G, Valsangkar B, Claeson M. Consensus on kangaroo mother care acceleration. *Lancet*. 2013;382(9907):e26-e27.
18. Guenther T, Moxon S, Valsangkar B, et al. Consensus-based approach to develop a measurement framework and identify a core set of indicators to track implementation and progress towards effective coverage of facility- based kangaroo mother care. *J Glob Health*. 2017;7(2):1-12.

19. United Nationals Children's Fund (UNICEF). *Committing to Child Survival: A Promise Renewed*. New York; 2013. Available from: www.apromiserenewed.org
20. Child Health Division Ministry of Health and Family Welfare Government of India. *Kangaroo Mother Care & Optimal Feeding of Low Birth Weight Infants*. New Delhi: Government of India; c2015.
21. Ministry of Health and Family Welfare Government of India. *India Newborn Action Plan (INAP)*. New Delhi: Government of India; c2014. Available from: https://www.newbornwhocc.org/INAP_Final.pdf
22. Save the Children, MCSP, Nigeria FMOH. *Assessment of Facility-Based Care of Small Newborns in Nigeria. Phase 1: Landscape Survey*. Abuja, Nigeria: Save the Children; c2017.
23. Mathewos B, Sitrin D, Valsankar B, et al. *Delivery of kangaroo mother care in Ethiopian facilities: results from a rapid assessment in three zones.* c2015. Available from: https://www.healthynewbornnetwork.org/hnn-content/uploads/KMC_Facilities_Brief_.pdf. c2015.
24. Ministerio de Salud Publica Republica Dominicana. *Alianza Nacional Para Acelerar La Reducción de La Mortalidad Materna e Infantil*. Santo Domingo: Ministerio de Salud Publica Republica Dominicana; c2019. Available from: https://www.msp.gob.do/web/Transparencia/documentos_oai/1065/mispas-daf-cm-2019-0179/10546/documento-marco-alianza-nacional-proceso-mispas-daf-cm-2019-0179.pdf.
25. Ministerio de Salud Publica Republica Dominicana. *Guía De Práctica Clínica Para La Atención Del Recién Nacido Prematuro*. Santo Domingo: Ministerio de Salud Publica Republica Dominicana;c2018.
26. Jamali QZ, Shah R, Shahid F, et al. Barriers and enablers for practicing kangaroo mother care (KMC) in rural Sindh, Pakistan. Ameh CA, ed. *PLoS One*. 2019;14(6):e0213225. doi:10.1371/journal.pone.0213225
27. World Health Organization, UNICEF. *Countdown to 2015: Decade of Tracking Progress for Maternal, Newborn and Child Survival.* Geneva:WHO; c2015.
